# Structural and Biochemical Studies of Human 4-hydroxy-2-oxoglutarate Aldolase: Implications for Hydroxyproline Metabolism in Primary Hyperoxaluria

**DOI:** 10.1371/journal.pone.0026021

**Published:** 2011-10-06

**Authors:** Travis J. Riedel, Lynnette C. Johnson, John Knight, Roy R. Hantgan, Ross P. Holmes, W. Todd Lowther

**Affiliations:** 1 Center for Structural Biology and Department of Biochemistry, Wake Forest School of Medicine, Winston-Salem, North Carolina, United States of America; 2 Division of Urology, Department of Surgery, Wake Forest School of Medicine, Winston-Salem, North Carolina, United States of America; University of Oulu, Finland

## Abstract

**Background:**

4-hydroxy-2-oxoglutarate (HOG) aldolase is a unique enzyme in the hydroxyproline degradation pathway catalyzing the cleavage of HOG to pyruvate and glyoxylate. Mutations in this enzyme are believed to be associated with the excessive production of oxalate in primary hyperoxaluria type 3 (PH3), although no experimental data is available to support this hypothesis. Moreover, the identity, oligomeric state, enzymatic activity, and crystal structure of human HOGA have not been experimentally determined.

**Methodology/Principal Findings:**

In this study human HOGA (hHOGA) was identified by mass spectrometry of the mitochondrial enzyme purified from bovine kidney. hHOGA performs a retro-aldol cleavage reaction reminiscent of the trimeric 2-keto-3-deoxy-6-phosphogluconate aldolases. Sequence comparisons, however, show that HOGA is related to the tetrameric, bacterial dihydrodipicolinate synthases, but the reaction direction is reversed. The 1.97 Å resolution crystal structure of hHOGA bound to pyruvate was determined and enabled the modeling of the HOG-Schiff base intermediate and the identification of active site residues. Kinetic analyses of site-directed mutants support the importance of Lys196 as the nucleophile, Tyr168 and Ser77 as components of a proton relay, and Asn78 and Ser198 as unique residues that facilitate substrate binding.

**Conclusions/Significance:**

The biochemical and structural data presented support that hHOGA utilizes a type I aldolase reaction mechanism, but employs novel residue interactions for substrate binding. A mapping of the PH3 mutations identifies potential rearrangements in either the active site or the tetrameric assembly that would likely cause a loss in activity. Altogether, these data establish a foundation to assess mutant forms of hHOGA and how their activity could be pharmacologically restored.

## Introduction

**Figure 1:**
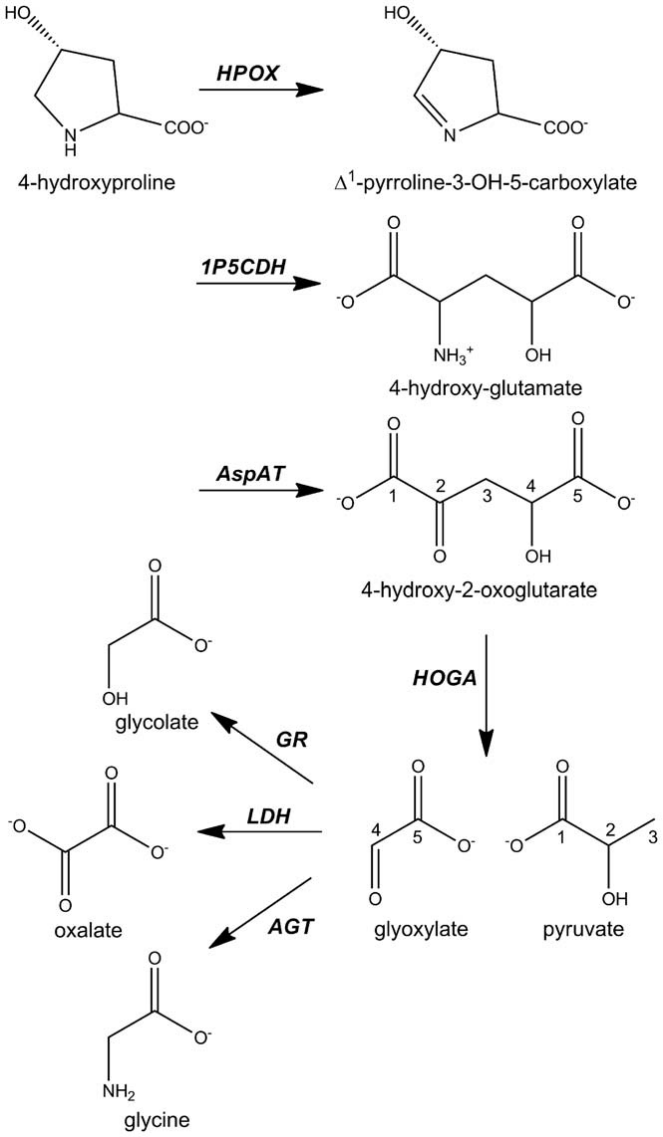
Metabolism of 4-hydroxyproline and glyoxylate. Four mitochondrial enzymes are responsible for 4-hydroxproline (4-Hyp) breakdown: hydroxyproline oxidase (HPOX), Δ^1^-pyrroline-5-carboxylate dehydrogenase (1P5CDH), aspartate aminotransferase (AspAT), and 4-hydroxy-2-oxoglutarate aldolase (HOGA). The terminal HOGA reaction cleaves 4-hydroxy-2-oxoglutarate (HOG) into pyruvate and glyoxylate. Glyoxylate is metabolized either to glycolate by glyoxylate reductase (GR) in the mitochondria and cytoplasm or to glycine by peroxisomal alanine-glyoxylate aminotransferase (AGT). AGT and GR are mutated within primary hyperoxaluria (type 1 and 2, respectively) patients resulting in the buildup of glyoxylate and its conversion by lactate dehydrogenase (LDH) to oxalate, a key component of kidney stones.

Glyoxylate is a highly reactive two carbon anion and traditionally thought to be produced through glycine and glycolate metabolism [1]–[4]. Recent studies, however, have demonstrated that significant glyoxylate and oxalate production can occur through the metabolism of 4-hydroxyproline (4-Hyp). It is estimated that 300–450 mg of 4-Hyp are produced each day from endogenous collagen turnover, and additional 4-Hyp is derived from the diet [5]. Since less than 30 mg of 4-Hyp is excreted in the urine, the majority of 4-Hyp is metabolized in the liver and kidney [6]. The degradation pathway for 4-Hyp (Figure 1) involves the step-wise action of four mitochondrial enzymes: hydroxyproline oxidase (HPOX), Δ^1^-pyrroline-5-carboxylate dehydrogenase (1P5CDH), aspartate aminotransferase (AspAT), and 4-hydroxy-2-oxoglutarate aldolase (HOGA, EC 4.1.3.16; also known historically as 2-keto-4-hydroxyglutarate aldolase and 4-hydroxy-2-ketoglutarate aldolase) [5], [7], [8]. The first three steps involve the oxidation of the 4-Hyp ring to Δ^1^-pyrroline-5-carboxylate, spontaneous ring opening and further oxidation, and the conversion of 4-hydroxy-glutamate to 4-hydroxy-2-oxogluarate (HOG). In the terminal reaction, HOG is cleaved by HOGA to produce pyruvate and glyoxylate. The resulting glyoxylate can be converted to glycine and glycolate by alanine-glyoxylate aminotransferase (AGT) and glyoxylate reductase (GR), respectively. Thus, in normal metabolism, the glyoxylate produced from 4-Hyp is readily detoxified.

In contrast, patients with primary hyperoxaluria (PH) can have dysfunctional AGT (PH type 1) or GR (PH type 2) [9]. As a result, glyoxylate levels rise and oxalate can be produced by lactate dehydrogenase (LDH) (Figure 1), ultimately resulting in the formation of calcium oxalate kidney stones [10], [11]. Therefore, the glyoxylate produced from 4-Hyp most likely exasperates the glyoxylate and oxalate levels of PH patients. A recent report using heterozygote mapping of a subset of individuals with an unclassified form of PH identified mutations in the *DHDPSL* gene [12]. Several additional mutations have subsequently been identified [13]. It was speculated that this gene is human HOGA, and a proposal was made to reclassify these patients as having PH type 3 (PH3). Direct evidence for the enzymatic activity of the *DHDPSL* gene product, however, was not obtained. Moreover, despite the greater than 40 years of work in delineating the 4-Hyp degradation pathway utilizing HOGA purified from bovine and rat mitochondria, the identity, oligomeric state, enzymatic activity, and crystal structure of human HOGA (hHOGA) have not been experimentally determined [8], [14]–[18].

The identification of HOGA mutations clearly highlights the need to better understand 4-Hyp metabolism in humans and raises many potential scenarios for increased oxalate formation. In one proposal the mutations found within hHOGA are thought to increase enzymatic activity and glyoxylate production, which would be consistent with the phenotypes observed in PH1–3 patients [9], [12], [13]. Alternatively, the HOGA variants could have decreased enzymatic activity, aberrant cellular targeting, and decreased stability. If any of these scenarios are correct, HOG could build up in concentration, but the physiological consequences are currently unknown. Experiments to determine the molecular basis for human HOGA substrate recognition and catalysis are therefore needed to evaluate the potential impact of mutations found in PH3 patients.

The work presented herein describes the identification of hHOGA by mass spectrometry of bovine HOGA purified from kidney mitochondria. The activity of recombinant hHOGA proves that it is indeed the aldolase identified in the genomic analysis of PH3 patients. The crystal structures of the apoenzyme and pyruvate-Schiff base intermediate complex are the first for any HOGA enzyme to date and reveal a tetrameric structure composed of a dimer of dimers as well as the identity of active site residues. Kinetic analyses of site-directed mutants have shown that Tyr168 and Lys196 are essential for catalysis, while Ser77, Asn78, and Ser198 appear to modulate substrate binding and catalysis. The mapping of the PH3 mutations suggests that some variants impact the active site architecture and others affect the oligomeric state. Altogether, these data provide new insights into the mechanism of hHOGA and a structural blueprint to assess the activity of mutant forms of hHOGA and their potential rescue.

## Results

The 4-Hyp catabolic pathway (Figure 1) has been delineated through the fractionation and characterization of mitochondrial lysates from the liver and kidney tissues of several mammals [8], [14]–[18]. The last step of the pathway, the cleavage of HOG to pyruvate and glyoxylate by HOGA, is of particular importance to PH patients where glyoxylate can be shunted to oxalate formation [4]. Thus, modulation of 4-Hyp degradation has the potential to ameliorate the production of glyoxylate from both endogenous and dietary sources of 4-Hyp [11], [19].

### Purification of bovine HOGA enables identification of human HOGA

**Figure 2:**
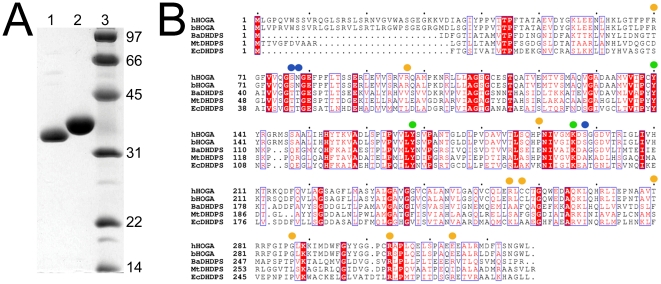
Identification of human HOGA and its sequence relationship to DHDPS enzymes. (A) SDS-PAGE analysis of bovine HOGA purified from kidney and human HOGA expressed in *E. coli*. Lane 1, bovine HOGA; lane 2, human HOGA with an N-terminal, 6-His tag; lane 3, protein molecular weight ladder indicated in kDa. (B) Sequence comparison of human HOGA with bovine HOGA (90.2% identity), *B. anthracis* DHDPS (31.1%), *M. tuberculosis* DHDPS (26.6%), and *E. coli* DHDPS (22.3%). Putative catalytic residues for hHOGA (Tyr140, Tyr168, and Lys196) are indicated by green circles. Blue circles indicate additional active site residues that were assessed by site-directed mutagenesis to evaluate their contribution to substrate specificity and catalysis. HOGA mutations identified within PH3 patients are denoted with orange circles [12], [13].

In order to identify hHOGA, mitochondria were purified from freshly dissected, bovine kidney cortex. Using a procedure adapted from Dekker *et al.*, bHOGA was purified using heat denaturation, ammonium sulfate fractionation, anion exchange chromatography, and size-exclusion chromatography (Figure 2A) [15]. During this process the fractions containing aldolase activity, i.e., production of glyoxylate from HOG synthesized in-house (see Experimental Procedures), were determined with a phenylhydrazine assay [14], [20], [21]. The HOG used in these studies is a racemic mixture. Previous studies have shown that the bovine kidney, bovine liver, and rat liver enzymes have equal activity against the *R*- and *S*-forms of HOG [15], [18], [22]. The specific activity of bHOGA increased with each purification step to 0.2 µmol glyoxylate min^−1^ mg^−1^, representing a ∼46,000-fold purification (data not shown). The final yield of bHOGA was 1.9 mg.

The bHOGA protein was digested with trypsin and the resulting peptide fragments analyzed by LC-MS/MS. Eight peptides, representing 43.7% sequence coverage, were found to match a bovine protein (Q0P5I5), annotated as a mitochondrial DHDPS-like protein (Figure 2B). Electrospray mass analysis of intact bHOGA revealed that the mature form of the protein begins at residue 26 (327 amino acids, 35,217 Da), and that the protein as purified contains no additional posttranslational modifications. The human HOGA homolog (NP_612422) was readily identified by a BLAST search, and an IRAT clone was obtained from Invitrogen. In order to clarify the many historical names used for HOGA, the gene and protein sequence databases have been updated to the following nomenclature *HOGA1* (NM_138413) and HOGA1, respectively.

### Human HOGA is related to bacterial DHDPS enzymes

**Figure 3:**
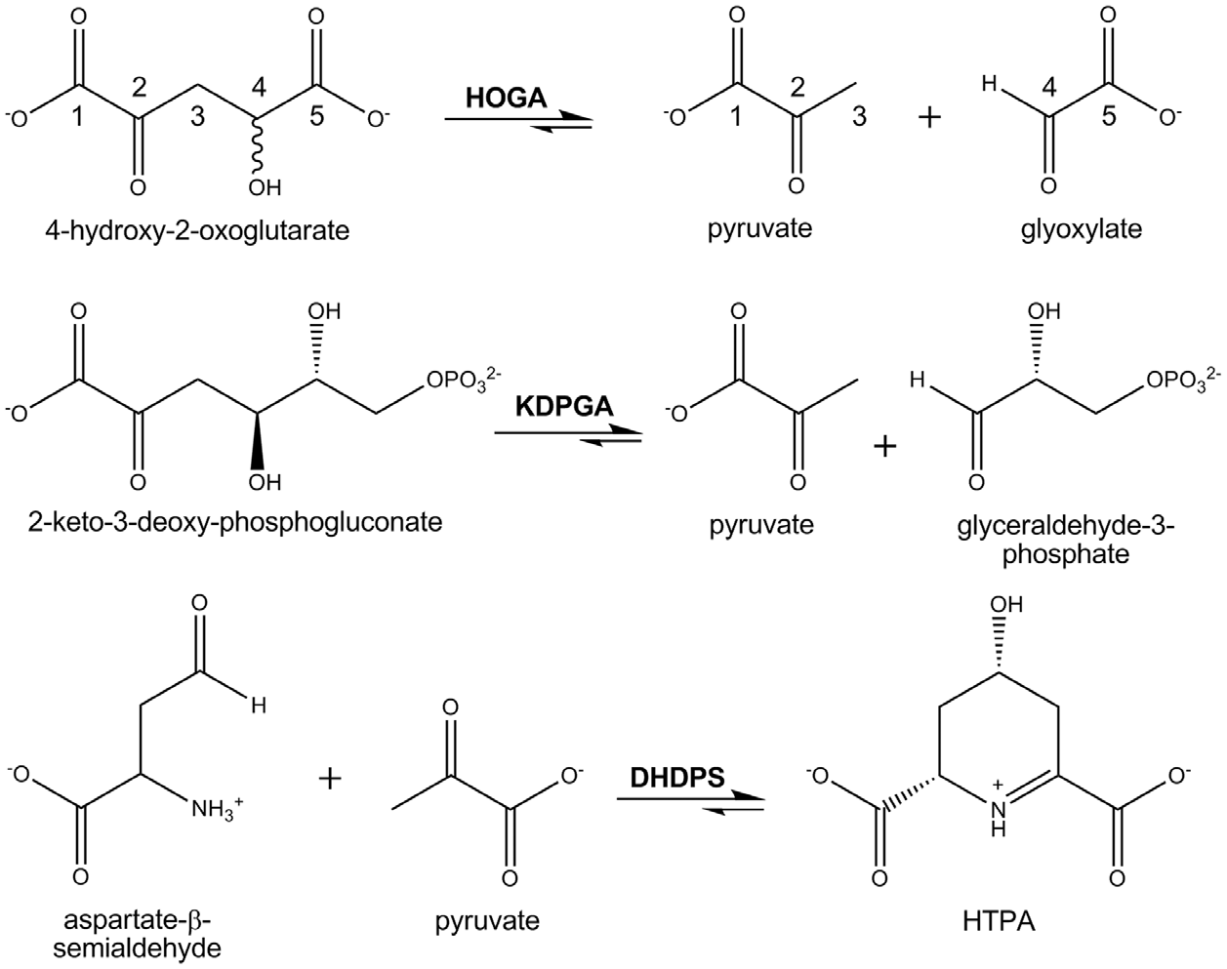
Comparison of the HOGA, KDPGA, and DHDPS reactions. The utilization of a Lys residue and Schiff base intermediate with pyruvate for catalysis is shared between the enzymes. However, the preferred reaction directions are opposite, i.e., HOGA and KDPGA perform cleavage reactions, while the DHDPS enzymes utilize a condensation reaction between pyruvate and (S)-aspartate-β-semialdehyde to form (4*S*)-4-hydroxy-2,3,4,5-tetrahydro-(2*S*)-dipicolinate (HTPA). The wavy bond on the 4-position of HOG indicates that the enzyme can cleave both the *S*- and *R*-forms of HOGA [15], [18], [22].

A survey of the literature reveals that the reaction catalyzed by HOGA (Figure 3) is most similar to the bacterial 2-keto-3-deoxy-phosphogluconate aldolase (KDPGA) and related bacterial aldolases [23]–[25]. These enzymes cleave their substrate and produce pyruvate as the common product. Biochemical and structural studies have shown that these enzymes function in a trimeric assembly (Figure S1, A and B). The active site contains conserved Arg17, Glu40, Lys129 and Thr156 residues (numbering for the *Thermatoga maritima* enzyme) that facilitate catalysis and formation of a Schiff base intermediate with the Lys residue [24]. Human and bovine HOGA both contain a conserved Lys196 (Figure 2B), but none of the other residues of the KDPGA active site motif.

A BLAST search revealed that hHOGA was actually most related to the dihydrodipicolinate synthase (DHDPS) from *Bacillus anthracis* (Figure 2B, 31.1% identity). The DHDPS enzymes perform a condensation reaction between pyruvate and (*S*)-aspartate-β-semialdehyde to yield (4*S*)-4-hydroxy-2,3,4,5,-tetrahydro-(2*S*) dipicolinate (HTPA), an unstable intermediate thought to undergo dehydration to (*S*)-2,3-dihydropicolinate [26]–[29]. Since this reaction is the first committed step in lysine biosynthesis, the DHDPS enzymes are an attractive antibacterial target. In contrast to KDPGA, these enzymes function as a tetrameric assembly (Figure S1, C and D). The active site of *E. coli* DHDPS contains five conserved residues, Thr44, Thr45, Tyr107, Tyr133, and Lys161, as illustrated by the pyruvate-Schiff base adduct structure (Figure S1D) [28]. The Tyr and Lys residues appear to be conserved in human and bovine HOGA: Tyr140, Tyr168, and Lys196 (Figure 2B).

### Solution behavior of recombinant hHOGA

In order to characterize the solution, kinetic, and structural properties of human HOGA, the gene was subcloned into the pET151/D-TOPO vector so that the mitochondrial targeting sequence was removed (i.e., residues 1–25). The protein was expressed recombinantly in *E. coli* and readily purified using an N-terminal His-tag and two additional column steps (Figure 2A). Removal of the His-tag by limited proteolysis did not affect the activity of the protein, but its removal did prevent aggregation at higher protein concentrations (data not shown). One peculiar observation from the purification warrants mention. During the size-exclusion Superdex 200 column run, the peak containing hHOGA eluted with a retention time midway between those calculated for trimeric and tetrameric assemblies (Figure S2), an apparent contradiction to the predicted tetramer based on the similarity to the DHDPS enzymes. Perhaps hHOGA has some intrinsic affinity for the Superdex 200 bead material despite the presence of 100 mM NaCl in the elution buffer.

**Figure 4:**
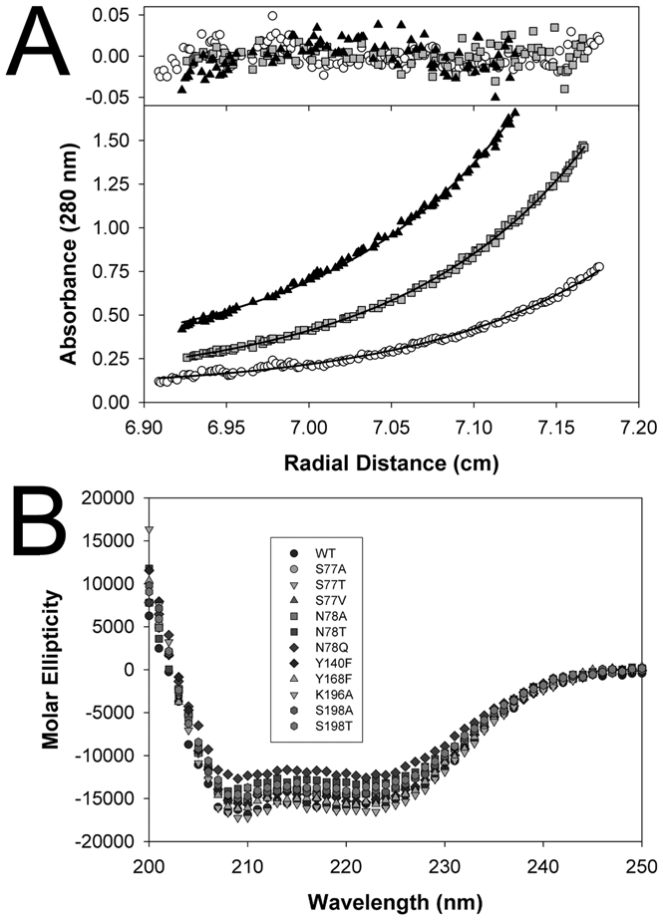
Characterization of recombinant hHOGA variants. (A) Representative analytical ultracentrifugation analysis of His-tag free HOGA at one rotor speed (10,500 rpm) and three different concentrations: black triangles, 1.25 mg ml^-1^; gray squares, 0.82 mg ml^−1^; open circles, 0.43 mg ml^−1^. The solid lines were obtained by a fit of these data and those obtained in a replicate experiment to a single ideal species model, which yielded MW  = 97,210±810; the residuals are shown above. (B) Circular dichroism analysis of all hHOGA variants within this study. Buffer conditions: 0.2 mg ml^−1^ HOGA variant, 20 mM HEPES pH 7.5, 100 mM NaCl, 25°C. Data are presented as molar ellipticity *vs.* wavelength.

Sedimentation equilibrium ultracentrifugation data were collected at three concentrations for the His-tag free protein (e.g., 0.43, 0.82, and 1.25 mg ml^−1^) and two rotor speeds (7,500 and 10,500 *g*) to investigate this discrepancy (Figure 4A). The simultaneous fit of these data and those obtained in a replicate experiment (0.45, 0.78 and 1.14 mg ml^−1^) using HETEROANALYSIS yielded a weight-average molecular weight, M_w_, of 97,200±810, a value 3-fold greater than that for a monomeric species, but considerably less than a tetramer (130,500). Further analysis using monomer-dimer-tetramer and dimer-tetramer models (HETEROANALYSIS and SEDPHAT, Figure S3) supports that a dimer-tetramer equilibrium is favored with a calculated K_d_ value of ∼60 µM [30]. The behavior of the His-tag free hHOGA was also analyzed by dynamic light scattering. A single mono-disperse peak (Figure S4) was obtained from solutions containing 0.25–25 mg mL^−1^ hHOGA. While the peak diameter (9.1±0.5 nm) is larger than that calculated for a globular trimer (6.7 nm) or tetramer (7.3 nm), asymmetry or increased hydration may contribute to this discrepancy. The prolate ellipsoidal, ring-like shape of the bacterial DHDPS enzymes (Figure S1C) and hHOGA, described in detail below, supports these possibilities.

### Crystal structure of human HOGA

In an effort to clarify the oligomeric state of hHOGA, to visualize the active site, and to determine the context of PH3 mutations, the structure of hHOGA was determined. A number of crystallization conditions were identified using commercial screens and the vapor diffusion technique. However, only one of these conditions upon optimization produced strongly diffracting crystals. A 2.5 Å resolution dataset was collected for the native apoenzyme at the National Synchrotron Light Source, beamline X25 (Table 1). The inclusion of 10 mM pyruvate during crystallization improved diffraction substantially and allowed for in-house data collection to 1.97 Å resolution. The crystals belong to space group P6_4_22 with the Matthew's coefficient indicating 2 molecules per asymmetric unit (a.s.u.) and 50% solvent content.

**Table 1:** Crystallographic data and refinement statistics.

Crystal	EMTS•hHOGA	Native hHOGA	Pyruvate•hHOGA
*Processing*			
Spacegroup	P6_4_22	P6_4_22	P6_4_22
Unit Cell Parameters			
* a, b, c (Å)*	141.9, 141.9, 107.6	142.1, 142.1, 108.1	141.2, 141.2, 108.4
* α, β, γ (°)*	90, 90, 120	90, 90, 120	90, 90, 120
Wavelength (Å)	1.54	1.50	1.54
Resolution^a^ (Å)	29.60–2.10 (2.18–2.10)	59.38–2.50 (2.59–2.50)	35.17–1.97 (2.04–1.97)
* R*_merge_ (%)	7.4 (33.4)	6.9 (26.7)	5.0 (34.0)
* R*_meas_ (%)	7.5 (34.4)	7.0 (27.0)	5.2 (35.4)
<I/σI>	25.2 (6.1)	28.3 (11.4)	20.8 (5.7)
Completeness (%)	99.9 (98.1)	100 (100)	99.9 (100)
Redundancy	36.7 (16.9)	41.3 (42.0)	13.8 (12.3)
Wilson B (Å^2^)	31.1	42.4	29.9
FOM			
Initial	0.51		
After density modification	0.70		
*Refinement*			
* R*_work_/*R*_free_ (%)	28.0/29.5^b^	21.2/24.6	19.6/21.6
No. of reflections (work/free)		21,644/1,170	43,028/2,287
No. atoms			
Protein		2,232	2,256
Ligands		24	30
Water		47	211
RMSD Bond Lengths (Å)		0.012	0.014
RMSD Bond Angles (°)		1.433	1.428
Average B-factor (Å^2^)			
Protein		42.3	28.9
Ligands		52.8	39.9
Solvent		41.4	39.7
Ramachandran analysis			
Favored regions		96.9%	98.0%
Allowed regions		3.1%	2.0%

a Numbers in parentheses are for the highest resolution shell. The same full resolution range was used for refinement.

b These values were obtained after solvent flattening and one round of CNS refinement.

Structure solution was first attempted using a monomer and dimer of *B. anthracis* DHDPS (PDB ID: 1XKY, 31% sequence identity) (Figure S1) as a molecular replacement search model within PHASER [31]. Despite trying a variety of permutations and adjustments to the search model to maximize homology (i.e., the use of CHAINSAW within CCP4i to prune non-conserved side chains and the removal of surface loops), this approach failed to provide a reasonable solution. Experimental phases were determined from crystals containing hHOGA pre-derivatized with the mercury-containing compound EMTS (Table 1). Using the anomalous Hg^2+^ signal, PHENIX identified 3 high-occupancy sites that yielded readily interpretable 2.1 Å resolution electron density maps (Figure S5A). The AutoBuild routine within PHENIX was able to build 97% of the structure. Interestingly, the packing of hHOGA within the unit cell (Figure S5B) supports only one molecule in the a.s.u. and a solvent content of 75%. The crystallographic symmetry, however, does generate a tetramer that rotates up the 3-fold screw axis, resulting in large solvent channels. The model was refined until the R_work/free_ values reached 28.0/29.5%, and then used as a molecular replacement search model to solve the structures of the apoenzyme and the Schiff base adduct with pyruvate. Of the 302 residues in the mature form of hHOGA, 296 were modeled for both structures. No electron density was observed for the first six residues of hHOGA and the three residues remaining from the affinity tag. The final structures for the apoenzyme and Schiff base adduct exhibited R_work/free_ values of 21.2/24.6% and 19.6/21.6%, respectively, and were of high overall quality, as indicated by the refinement statistics in Table 1.

**Figure 5:**
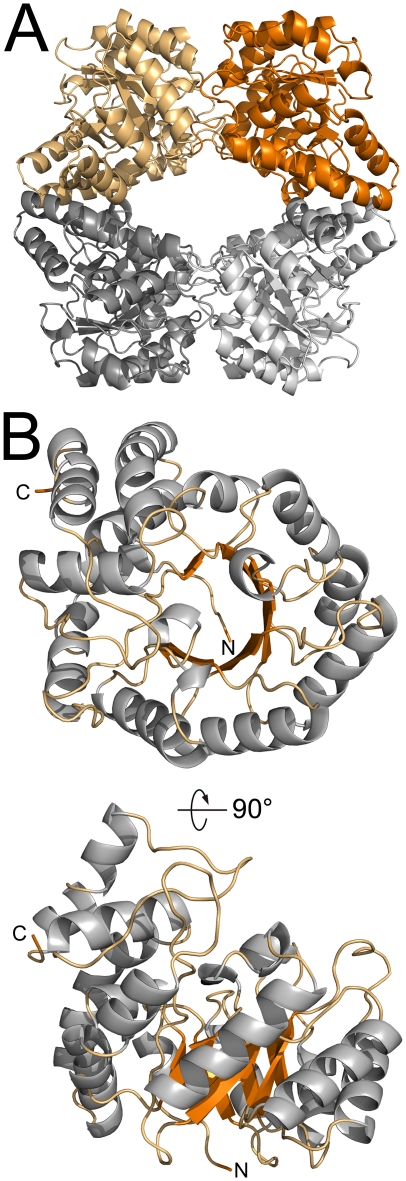
Structure of human HOGA. (A) Homo-tetrameric organization of hHOGA. The tetramer consists of a dimer of dimers (orange and gray). (B) The hHOGA monomer. Two orthogonal views illustrate the overall architecture, TIM barrel fold, and C-terminal helical bundle. Coloring is as follows: gray, α-helices; orange, β-strands; light-orange, loop regions.

As mentioned briefly above, the crystallographic symmetry of the hHOGA crystals generate a tetrameric assembly (Figure 5A and Figure S5) containing a dimer of dimers. Residues 26–259 form an (α/β)_8_ TIM barrel domain, while residues 260–327 form a three-helical bundle at the C-terminus (Figure 5B). The dimer-dimer or “loose” interface (orange versus gray in Figure 5A) primarily involves the interactions between the four helical bundles, as described within the DHDPS literature [32]–[34]. In contrast, the active site is located at the C-terminal end of the TIM barrel domain near the monomer-monomer interface of the “tight” dimer (colored different shades of orange and gray in Figure 5A). This interface involves many surface loops, one of which protrudes into the active site of the adjacent monomer.

**Figure 6:**
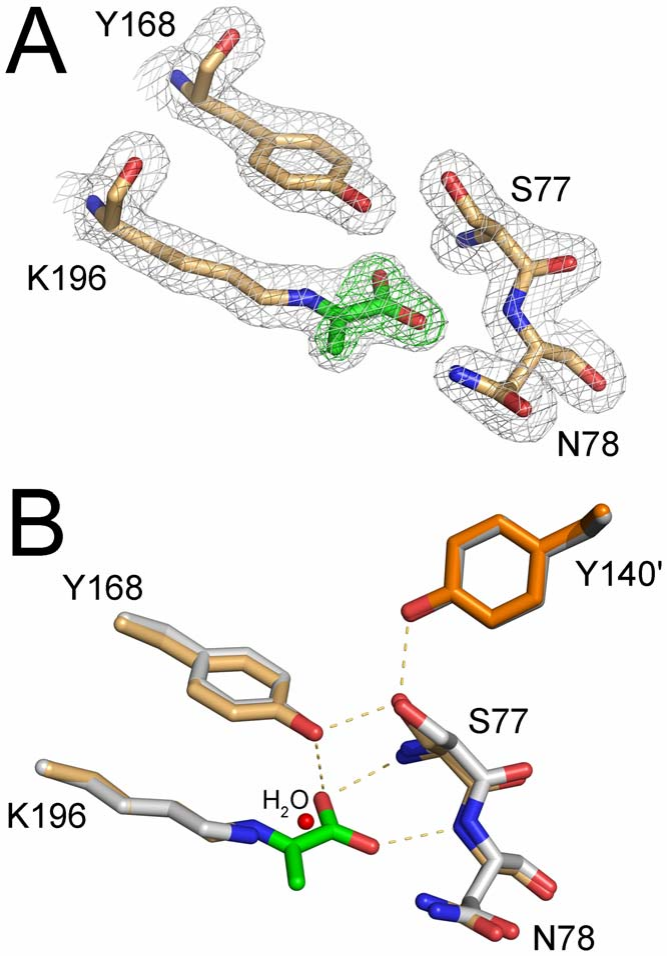
Active site structure of hHOGA. (A) Pyruvate-Schiff base complex. The 1.97 Å resolution, 2*F_o_-F_c_* electron density map is shown in gray and contoured to 1.5 σ. The pyruvate molecule is highlighted by a green *F_o_-F_c_* omit map contoured to 6.4 σ. Atom colors are as follows: blue, nitrogen; light-orange, carbon atoms for hHOGA; green, carbon atoms for pyruvate; red, oxygen. (B) Comparison to the unliganded active site. Coloring for pyruvate bound hHOGA is the same as in (A). White coloring is used for unbound hHOGA. Tyr140′ from the adjacent monomer are colored in a darker shade of orange and gray, respectively. A water molecule adjacent to Lys196 is depicted as a red sphere in the unbound hHOGA structure.

An omit *F_o_-F_c_* map contoured to 6.4 σ shows clear continuous electron density for the imine Schiff base adduct between the ε-amino group of Lys196 and pyruvate (Figure 6A). In this binding mode, hydrogen bonding interactions are observed between the hydroxyl group of Tyr168 and the O^(1B)^ carboxylate oxygen atom of pyruvate (Figure 6B). Additional hydrogen bonding interactions are present between the backbone amide nitrogen atoms of Ser77 and Asn78 and the O^(1B)^ and O^(1A)^ atoms of pyruvate, respectively. A network between Ser77, Tyr168, and Tyr140' from the adjacent monomer is also formed. A superposition of the apoenzyme structure with the pyruvate adduct (RMSD  = 0.19 Å for C_α_ atoms) reveals no significant structural changes to the active site other than a water molecule within hydrogen bonding distance to Lys196 (Figure 6B).

### Assessment of putative catalytic residues

**Figure 7:**
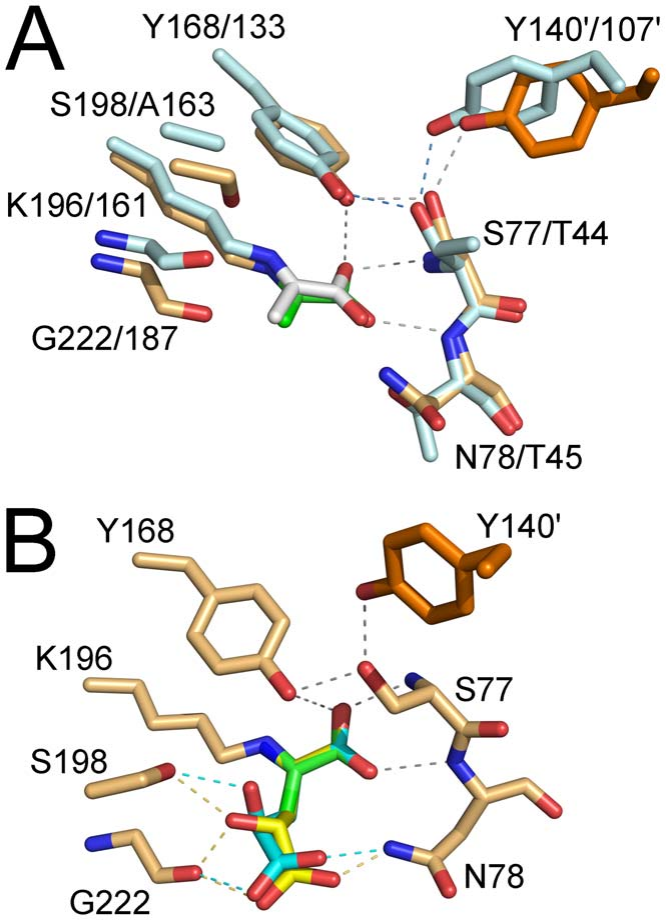
Structural comparisons between hHOGA and *E. coli* DHDPS. (A) Superposition of hHOGA and *E. coli* DHDPS (PDB ID: 3DUO) pyruvate-bound active sites (RMSD  = 0.19 Å for C_α_ atoms) [28]. hHOGA coloring is the same as in Fig. 6. DHDPS carbon atoms are colored in pale cyan and cyan (adjacent subunit). Carbon atoms for pyruvate bound to DHDPS are colored gray. Hydrogen bonds are shown using dashed lines for hHOGA (gray) and DHDPS (blue). Amino acid residues for the two enzymes are indicated in the following order: hHOGA/DHDPS. (B) Model of the HOG•hHOGA complex. HOG carbon atoms and putative hydrogen bonds to hHOGA are colored yellow (*R*-form) and cyan (*S*-form).

A superposition of hHOGA and *E. coli* DHDPS monomers in complex with pyruvate (Figure 7A) confirms the overall structural homology (RMSD  = 1.7 Å for C_α_ atoms). The Schiff base adducts and the surrounding active site residues also overlay well. The same hydrogen-bonding network involving the two Tyr residues (Tyr140′/107′ from the adjacent monomer and Tyr168/133; the second residue number is for *E. coli* DHDPS), two backbone amide nitrogen atoms (Ser77/Thr44 and Asn78/Thr45), and the side chain of Ser77/Thr44 appear to be maintained with minor adjustments. Ser77 of hHOGA is present within a conserved G-X-X-G-E loop motif, where X is a variety of amino acids (Gly76-Ser77-Asn78-Gly79-Glu80 in hHOGA). This motif is found within all DHDPS enzymes [26]–[29], [35]. Interestingly, Ser77 interacts with both Tyr168 and Tyr140'.

In order to test whether hHOGA utilizes the same catalytic residues as the DHDPS enzymes, site-directed mutants for Tyr140, Tyr168, Lys196 and Ser77 (Figure 7A) were generated and purified. Circular dichroism analysis (Figure 4B) showed that all the hHOGA variants had similar spectra. Secondary structure index calculations showed that each mutant agrees within 10% of wild-type hHOGA, supporting that these mutations did not cause gross structural changes. The kinetic parameters for these variants were readily determined using a coupled assay that relied upon the consumption of NADH and the conversion of pyruvate to lactate by LDH (Figure S6).

The Y140F mutant of hHOGA, evaluating a residue that protrudes into the active site from the adjacent monomer (Figure 7A), showed activity (Table 2) directly comparable to the WT enzyme with *k*_cat_, *K*_M_ and *k*_cat_/*K*_M_ values of 5.2 s^−1^, 8 µM, and 650 mM^−1^ s^−1^, respectively. This observation contrasts with the comparable Y107F variant of *E. coli* DHDPS that resulted in an 12-fold decrease in *k*_cat_ [35]. The Y168F and K196A hHOGA variants exhibited no enzymatic activity, even with 1,500 nM enzyme and 450 µM HOG present in the reaction. These significant losses in activity are similar to the 177-fold and 1,127-fold decreases in *k*_cat_ observed for the same variants of *E. coli* DHDPS [35]. The S77A variant exhibited a 2-fold decrease in *k*_cat_ and a nearly 8-fold increase in the *K*_M_ value for HOG compared to the WT enzyme (Table 2), resulting in an 20-fold reduction in the catalytic efficiency (*k*_cat_ */K*_M_ *).* The mutation of Ser77 to Thr, in order to mimic the DHDPS enzymes, also caused a significant loss in activity. A 50-fold decrease in *k*_cat_ */ K*_M_, with a comparable reduction and increase in *k*_cat_ and *K*_M_, was observed, respectively. A third mutant at this residue, S77V, was analyzed to simulate the bulk of the Thr residue, but without the hydroxyl group functionality. This variant exhibited reduced activity with kinetic parameters quite similar to the S77T variant.

**Table 2:** Kinetic parameters.

	*k*_cat_ (sec^-1^)	*K*_M_ (µM)	*k*_cat_ */K*_M_ (M^-1^ sec^-1^) × 10^3^	Relative *k*_cat_	Relative *k*_cat_ */K*_M_
**WT**	6.1±0.1	11±1	555±50	1.00	1.00
**S77A**	2.5±0.1	84±5	30±2	0.41	0.05
**S77T**	0.8±0.1	58±8	14±2	0.13	0.02
**S77V**	1.2±0.1	82±7	15±1	0.20	0.02
**N78A**	7.6±0.1	66±4	115±7	1.25	0.20
**N78T**	4.0±0.1	16±1	250±16	0.66	0.45
**N78Q**	6.2±0.1	281±14	22±1	1.02	0.04
**Y140F**	5.2±0.1	8±1	650±81	0.85	1.22
**Y168F**	–a	–	–	–	–
**K196A**	–	–	–	–	–
**S198A**	2.4±0.1	44±2	55±3	0.39	0.09
**S198T**	7.8±0.1	77±5	101±7	1.28	0.18

a No activity measured with the addition of 1.5 µM enzyme to the reaction containing 450 µM substrate.

### Assessment of residues involved in substrate specificity

In an effort to understand how hHOGA binds HOG and the apparent lack of stereospecificity for the 4-hydroxyl moiety, we generated a model for how each stereoisomer of the Lys196-HOG Schiff base intermediate could bind within the active site (Figure 7B). The (*R*)- and (*S*)-stereoisomers of HOG were generated with the Dundee PRODRG2 server and energy minimized [36]. The pyruvate portion of each HOG molecule was superimposed onto the pyruvate molecule of the crystal structure (Figure 6). Only minor rotational adjustments about the C3-C4 and C4-C5 bonds (Figure 1) were necessary to optimize the hydrogen-bonding distances and geometry for interactions with the side chain of Asn78, Ser198, and the backbone carbonyl group of Gly222. It is important to note that Asn78 and Ser198 of hHOGA are substituted within *E. coli* DHDPS active site to Thr45 and Ala163, respectively, (Figure 7A). The resulting models support that hHOGA can readily bind both HOG stereoisomers and highlights the potential importance of Asn78, Ser198, and Gly222 for the binding of HOG.

Site-directed mutants of Asn78 and Ser198 were evaluated to test the importance of these side chains to the hHOGA reaction. Circular dichroism spectra of all variants were similar to the WT enzyme (Figure 4B). While replacing the side chain of Asn78 with a methyl group (N78A) caused no change in turnover (*k*_cat_) (Table 2), a 6-fold increase in the *K*_M_ yielded a 4.8-fold reduction in *k*_cat_ */K*_M_ relative to the WT enzyme. Interestingly, the N78T variant, which mimics the DHDPS enzymes, exhibited a 1.5-fold decrease in *k*_cat_, but retained a WT-like *K*_M_ value. Asn78 was also mutated to Gln to test whether extending the side chain by one carbon atom would affect catalysis. This mutation caused a drastic loss in catalytic efficiency (*k*_cat_ */K*_M_), primarily due to the 25-fold increase in the *K*_M_ value for HOG. The equivalent residue to Ser198 of hHOGA in DHDPS is an Ala. This side chain difference makes sense, as the DHDPS substrates do not contain a hydroxyl group comparable to the 4-hydroxyl group of HOG. Mutating Ser198 to Ala caused a 2.5-fold decrease in *k*_cat_ and a 4.2-fold increase in *K*_M_, resulting in a 10-fold decrease in *k*_cat_ */K*_M_ compared to WT hHOGA. The S198T variant, which should be able to interact with the 4-hydroxyl group of HOG, exhibited a 7-fold increase in *K*_M_, but a *k*_cat_ value comparable to WT.

## Discussion

The catabolism of 4-Hyp derived from endogenous and dietary sources contributes to the levels of glyoxylate, glycolate, and glycine (Figure 1) within liver and kidney cells [11], [19]. The synthesis and breakdown of these metabolites is altered in PH patients and can lead to conversion of glyoxylate to oxalate and the subsequent formation of kidney stones and in extreme cases kidney failure [4]. Therefore, the pharmacologic or genetic manipulation of 4-Hyp catabolism and glyoxylate formation holds promise for these patients, but the human enzymes have not been biochemically characterized. Historically, the 4-Hyp catabolic pathway has been delineated primarily from the analysis of mitochondrial extracts from the kidneys and livers of rats and cows [8], [14]–[18]. In this study, we purified HOGA from bovine kidney mitochondria and used mass spectrometry to determine its protein sequence and corresponding gene sequence (Figure 2). With this information in hand, the gene for the human HOGA protein, *HOGA1*, was identified, cloned into an *E. coli* expression vector, and the protein purified to homogeneity. Recombinant hHOGA readily cleaves HOG yielding two products, glyoxylate and pyruvate (Figure 3 and Figure S6), thus demonstrating for the first time a direct gene-protein-activity correspondence for hHOGA.

From a chemical perspective, the reaction catalyzed by hHOGA is reminiscent of KDPGA from *E. coli* (Figure 3) and related bacterial enzymes that function in a trimeric assembly of monomers exhibiting the (β/α)_8_-barrel fold (Figure S1A and B) [23]–[25]. These enzymes cleave similar substrates, utilize a Lys-Schiff base intermediate, and produce pyruvate as one of the products. Therefore, we were surprised when sequence analyses showed that hHOGA is actually related to the bacterial DHDPS enzymes (Figure 2*B*) involved in lysine biosynthesis [26]–[29]. The latter enzymes also utilize a conserved Lys residue and function as a tetrameric, (β/α)_8_ assembly (Figure 2 and Figure S1C and D).

Our analysis of recombinant hHOGA by sedimentation equilibrium ultracentrifugation, size-exclusion chromatography, and dynamic light scattering (Figures 4A, S2 and S4) suggests that hHOGA may exhibit unusual solution behavior, with the data supporting trimeric, tetrameric, and potentially even higher-order assemblies. Fits of the ultracentrifugation data to monomer-dimer-tetramer and dimer-tetramer equilibria support the latter (Figure S3). A range of oligomeric states (72–144 kDa; i.e., dimer to tetramer) have also been observed for the rat and bovine enzymes, although the bovine enzyme was found to be a tetramer [14], [15], [18], [22]. A similar situation occurred with the structurally and functionally-related N-acetylneuraminate lyase enzymes that were first thought to be trimers, but were later found to be tetramers once the crystal structure was determined [37], [38]. Moreover, it is possible that hHOGA may exist as a mixture of dimers and tetramers (i.e., dimer of dimers, Figure 5A) in solution at low concentration, an observation found for some DHDPS variants [34], [39]. Altogether, these observations and the new crystal structures of hHOGA (Figures 5 and 6) support that HOGA is most likely functional as a tetramer.

The connection between hHOGA and the DHDPS enzymes is quite intriguing, as the favored reaction directions are opposite (Figure 3). DHDPS enzymes preferentially perform a condensation reaction [26]–[29]. In contrast, the equilibrium constant for the cleavage reaction for bovine and rat HOGA, determined using ^14^C-labeled substrates, ranges from 9.0–11.9 [18], [40]. These hHOGA homologs, however, can perform a condensation reaction between glyoxylate and pyruvate and several pyruvate analogs, although the reaction rates are 1,000–10,000-fold slower [14], [41], [42]. Nonetheless, it is possible that hHOGA could be modified to be useful for carbon-carbon bond formation and the synthesis of complex molecules, as has been observed for the native and various engineered forms of KDPGA and other bacterial enzymes [23], [43]–[45]. One significant difference for HOGA, however, is that it is not stereospecific for either the *R*- or *S*-forms of HOG [15], [18], [22]. It is of interest to note that the tetrameric 2-keto-3-deoxygluconate aldolases from *Sulfolobus solfataricus* and *Thermoproteus tenax* are additional examples of distantly-related, DHDPS-like enzymes that can perform a cleavage reaction similar to HOGA [46], [47].

hHOGA shares several active site residues with *E. coli* DHDPS (Figure 2B). Tyr107, Tyr133, and Lys161 of *E. coli* DHDPS correspond to Tyr140, Tyr168, and Lys196 of hHOGA (Figure 7A). Tyr107 and Tyr140 protrude into the active site of an adjacent monomer within the tetramer. Mutational analyses of *E. coli* DHDPS support that Tyr107, Tyr133, and Thr44, a residue unique to DHDPS discussed in more detail below, facilitate catalysis by functioning as a proton shuttle between the active site and solvent [35]. The loss in activity for the Y168F and Y133F variants of hHOGA and *E. coli* DHDPS, respectively, is consistent with the role of this residue to function as a general acid/base in the proton shuttle. The 12-fold loss in activity for the Y107F *E. coli* DHDPS variant, however, contrasts with the wild-type activity of the equivalent Y140F hHOGA mutant (Figure S6), supporting that this residue does not play as essential a role as in the DHDPS enzymes [35]. Lys161 forms a Schiff base with pyruvate, and the mutation of Lys161 to Arg and Ala in this DHDPS resulted in a 300–800-fold loss in activity [48]. The Schiff base intermediate observed within the hHOGA crystal structure (Figure 6) and the inactivity of the K196A variant (Table 2) support that this residue plays the same functional role in hHOGA catalysis.

The crystal structure of hHOGA in complex with pyruvate (Figures 6A and 7A) illustrates how the highly conserved G-X-X-G-E motif fulfills the same carboxylate-binding role via two backbone amide nitrogen atoms. This motif contains Thr44-Thr45 in the X-X positions for *E. coli* DHDPS, while hHOGA contains Ser77-Asn78. Importantly, the side chain of Thr44/Ser77 interacts with Tyr133/Tyr168 (Figure 7A). The modulation of all kinetic parameters for site-directed variants of these residues for both enzymes is consistent with their role in substrate binding and catalysis. For example, it was originally thought that mutating Ser77 to Thr would maintain a WT level of hHOGA activity because of the conservation of the hydroxyl functional group. The S77T variant, however, caused a significant decrease in *k*_cat_ and *k*_cat_ */K*_M_ values (Table 2). Similar findings have been observed for the DHDPS enzyme where the Thr was mutated to Ser (*k*_cat_ reduced 12-fold) [49]. Mutation of Ser77 to Ala and Val also exhibited reduced *k*_cat_ and *k*_cat_ */K*_M_ values compared to WT hHOGA, further supporting the importance of this hydroxyl group in the proposed proton relay. As noted above, however, hHOGA differs from *E. coli* DHDPS in that Tyr140 from the adjacent monomer appears not to be necessary for the shuttling of protons.

With the binding mode of the pyruvate-Schiff base intermediate in hand, we sought to model the interaction of the HOG-based imine intermediate in order to identify additional residues that could influence substrate binding and catalysis. Both the *S*- and *R*-forms of HOG were readily docked into the active site (Figure 7B). Several putative interactions were readily identified: the side chain of Asn78 with the terminal carboxyl group at the 5-position of HOG (Figure 1), the side chain of Ser198 with the 4-hydroxyl group, and a bifurcated interaction between the backbone carbonyl oxygen atom of Gly222 and the 4-hydroxyl group and one of the terminal carboxyl oxygen atoms of HOG. Asn78 of hHOGA was mutated to Ala, Thr, and Gln to evaluate its potential role. Interestingly, the Ala variant exhibited a similar *k*_cat_ value but a 6-fold increase in *K*_M_ compared to WT hHOGA (Table 2). The Thr variant, which mimics Thr44 of *E. coli* DHDPS, exhibited a significant decrease in *k*_cat_, but a WT-like *K*_M_ value. Extension of the side chain to Gln, however, caused a dramatic loss *k*_cat_/*K*_M_ due to a 25-fold increase in the *K*_M_ value for HOG. These results are consistent with severely compromised activity of comparable DHDPS variants [35], [50], further supporting an important role for Asn78 in substrate binding and catalysis.

The presence of Ser198 and its putative interaction with the 4-hydroxyl group of HOG are unique to HOGA enzymes. *E. coli* DHDPS has an Ala163 at the equivalent position (Figure 7A). The S198T variant, which should be able to interact with the 4-hydroxyl group of HOG, exhibited a 7-fold increase in *K_M_*, but a *k*_cat_ value (Table 2) comparable to the WT enzyme. The Ala variant exhibited decreased catalytic activity, increased *K*_M_ values, and decreased overall efficiency. These data, the conservation of Ser198 and Asn78 for the HOGA enzymes, and the lack of these residues in the DHDPS enzymes are also consistent with the differences in the substrate size and chemical structure beyond the core pyruvate molecule. Further support for this notion comes from the mutational analysis of Arg138 of *E. coli* DHDPS, which has been shown to be important for binding of the second substrate, (*S*)-aspartate-β-semialdehyde, near Tyr133 and Tyr107 and may also function to stabilize the proton relay [50]. Arg138 is substituted by Asn173 in hHOGA. Altogether, these data support the unique interactions of Asn78 and Ser198 with HOG.

**Figure 8:**
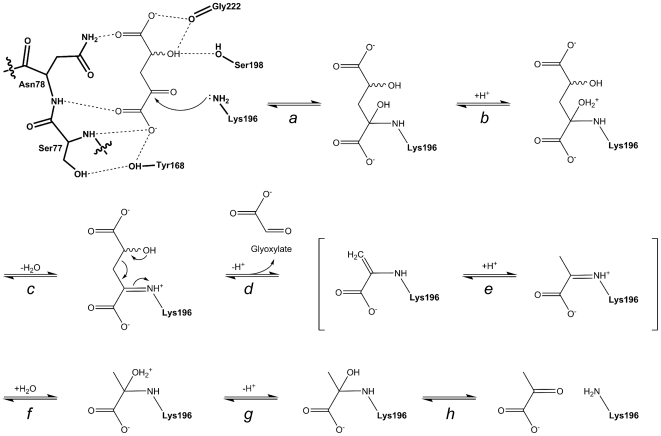
Proposed mechanism for hHOGA catalysis. The enzyme residues are highlighted in bold. See text for details.

Based on the new structural and kinetic data presented (Figures 5–6 and Table 2), as well as the comparisons to the well-studied structures (Figure 7) and mechanisms of the DHDPS and N-acetylneuraminate lyase enzymes, a classical, type 1 aldolase reaction mechanism for hHOGA can be proposed (Figure 8). HOG binds within the hHOGA active site so that the 2-oxo carbonyl group is adjacent to Lys196, the adjacent carboxylate binds to the backbone amide nitrogen atoms of Ser77-Asn78, the 4-hydroxyl group interacts with the unique residue Ser198, and the distal carboxylate interacts with the backbone carbonyl of Gly222 and the side chain of Asn78. The ε-amino group of Lys196, presumably exhibiting a lower p*K_a_* value, stages a nucleophilic attack (step *a*) on the 2-oxo group of HOG to form a zwitterionic intermediate that readily converts to a carbinolamine. The addition of a proton from the proton relay involving residues Tyr168 and Ser77 (step *b*) leads to dehydration (step *c*) and formation of the Schiff base with Lys196. This intermediate rearranges (step *d*) to release a proton and the first product of the reaction, glyoxylate. The resulting enamine can tautomerize (step *e*) to form the pyruvate-based imine. The addition of a water molecule, loss of a proton, and collapse of the resulting carbinolamine (steps *f*–*h*) ultimately leads to the release of pyruvate so that the catalytic cycle can begin again.

During the course of this study, 15 previously unclassified PH patients were shown to exhibit six mutations in a putative DHDPS-like protein [12]. Further analyses have identified four additional mutations in PH patients [13]. A series of assumptions were made to suggest that the DHDPS-like protein performs the same function as hHOGA. They proposed that the enzymes were the same based on the bovine enzyme being involved in glyoxylate metabolism, performing a lyase reaction, and being localized primarily in the mitochondria of liver and kidney [12]. However, no experimental data confirming any of these attributes for the human enzyme was available at that time. A direct link between the rat and bovine HOGA activity to the *DHDPSL* gene was also not possible, as the sequences for the neither the gene nor protein were determined. Nonetheless, it was proposed that these patients be reclassified as PH3. The experimental data presented herein definitively shows that hHOGA is indeed the enzyme responsible for the cleavage of HOG to glyoxylate within the 4-Hyp degradation pathway.

**Figure 9:**
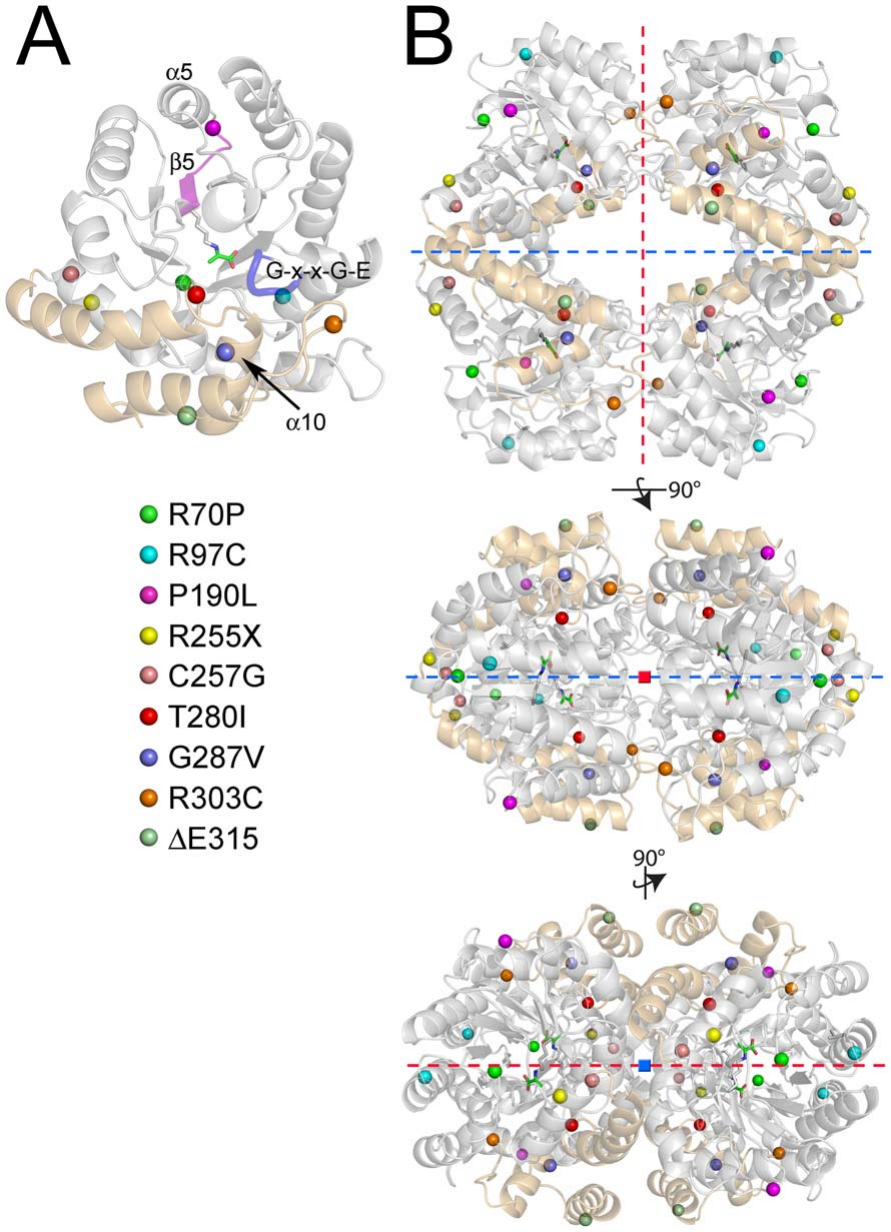
Location of mutations identified within hHOGA of PH3 patients. (A) Close-up view of one hHOGA monomer. The cartoon of the protein has been made partially transparent so that the PH3 mutations (spheres; see legend) are highlighted. The G-X-X-G-E motif is illustrated as a blue cartoon tube. The portion of the protein structure leading from the P190L variant to the active site Lys196-pyruvate adduct (stick rendering) is shown in magenta. (B) Three orthogonal views of the hHOGA tetramer illustrate the proximity of the mutations to the monomer-monomer (red dashed line) and dimer-dimer interfaces (blue dashed line). The red and blue squares indicate when an axis is projecting out toward the viewer.

Belostotsky *et al.* have also hypothesized that the mutation of HOGA in PH3 patients results in an increase in glyoxylate and oxalate production, despite having functional AGT and GR proteins (Figure 1) that should be able to handle the level of glyoxylate produced from 4-Hyp [12]. An equally attractive alternative hypothesis for why mutations in HOGA cause PH3 is that the activity of HOGA is partially or completely attenuated. In this scenario, the HOG, and perhaps the preceding metabolites, may increase in concentration and be ultimately converted to glyoxylate and pyruvate by an enzyme(s) that remains to be identified. An imbalanced metabolism may result in much more of the glyoxylate being converted to oxalate rather than glycolate, as occurs normally. A mapping of the nine exonic mutations identified to date onto the hHOGA structure (Figure 9) suggests some potential reasons for a loss in catalytic activity.

Four of the hHOGA variants can be categorized as potential active site disrupting mutants. The mutation of Pro190 to Leu, located in the turn connecting α-helix 5 to β-strand 5 where the essential catalytic residue Lys196 and substrate-positioning residue Ser198 reside (Figure 9A, highlighted in magenta), most likely disrupts the correct positioning of these key functional residues. The R70P mutation is located on the underside surface of the molecule upstream from the G76-X-X-G-E80 pyruvate-binding motif (Figure 9A, blue coil). The introduction of a Pro at this position could affect the register of Ser77 and Asn78 and therefore alter substrate binding and catalysis (Figures 7B and 8). The G287V and R303C variants may also disrupt the positioning of the G-X-X-G-E motif. Gly287 is located in a turn at the start of α-helix 10 and just two residues away from Lys289, which is involved in a salt bridge interaction with Glu80. Arg303 also participates in a salt bridge, but with another conserved residue, Glu88, downstream (not shown). The consequences of the R97C and T280I mutations are not obvious as they are not near the active site or the interface between the monomers within the tetramer.

The remaining mutations, R255X, C257G and ΔE315, are more distant from the hHOGA active site and most likely affect the overall folding and equilibrium between dimer and tetramer. R255X is a nonsense mutation that would truncate the entire C-terminal, three-helical bundle, a key feature of the loose dimer-dimer interface (Figure 5A and 9B). The C257G variant is located nearby and may also affect the structural transition between the TIM barrel and the three-helical bundle. The ΔE315 variant results in the deletion of one Glu residue in a triplet Glu motif present in the C-terminal helix. While this variant maintains the protein coding register, it could potentially alter the entire structure of this helix, also resulting in the disruption of the dimer-dimer interface. Further support for tetramer destabilization in these PH3 variants comes from the analysis of *E. coli* DHDPS variants. The variants were engineered to disrupt the tight monomer-monomer interaction involving Tyr107 and the loose dimer-dimer interface [32]–[34]. Mutation of Tyr107 to Trp resulted in a mixture of monomer and tetramer in solution and a loss in activity and thermal stability. The addition of the L197D mutation, a residue at the dimer-dimer interface, completely shifted the protein to the monomer, led to a further loss in activity, and resulted in an increased propensity for aggregation. Removal of the entire C-terminal, 3-helical bundle also compromised activity and quaternary structure.

In summary, human HOGA has been identified by mass spectrometry of the bovine enzyme from kidney mitochondria. The enzyme readily cleaves HOG and exhibits unusual solution properties, but most likely functions in solution as a tetramer due to its sequence and structural conservation with bacterial DHDPS enzymes. The crystal structure of the enzyme in complex with pyruvate has enabled the modeling of the HOG complex and led to the identification of key active site residues. Kinetic analyses confirm the necessity for Tyr168 and Lys196 in catalysis. Moreover, key roles for Ser77, Asn78 and Ser198 have been ascribed. The location of mutations found in PH3 patients suggest that some mutations directly affect the active site architecture while others may affect the oligomeric state. As a result, this study lays the foundation on which to assess the effect of HOGA mutations observed within PH3 patients on enzyme activity, protein folding, and subcellular targeting, all potential functional consequences previously observed in PH1 and PH2 patients [51], [52]. Experiments to evaluate these possible scenarios are currently in progress.

## Materials and Methods

### Synthesis of 4-hydroxy-2-oxoglutarate

The substrate for HOGA was synthesized via a base-catalyzed aldol condensation between oxaloacetate and glyoxylate [14], [20]. Briefly, a 25 mL 0.1 M KOH solution containing 0.92 g glyoxylate and 1.32 g oxaloacetate (1∶1 molar ratio) was adjusted to pH 12 using 10 M KOH and allowed to sit overnight. The solution became light yellow during this process and was tested using a phenylhydrazine assay to confirm that all of the glyoxylate had been consumed [21]. The pH was gradually decreased to ∼pH 5.5 using concentrated HCl; the solution bubbled vigorously during this decarboxylation step. The solution was quickly concentrated to ∼10 mL using a rotary evaporator set to 40°C; a higher temperature leads to degradation of HOG. HOG was then passed over a formate-form AG 1X8 column (BioRad, 200–400 mesh) equilibrated with water in order to remove chloride ions. The resulting solution was concentrated and passed over a H^+^-form AG 50W-X8 column (BioRad, 200–400 mesh) to remove potassium ions. The eluent was diluted with water and concentrated by roto-evaporation several times to remove formate. The final solution was concentrated to ∼5–6 mL, aliquoted, and stored at −20°C. A fresh aliquot was thawed for each experiment and kept on ice. The concentration of the HOG stock was determined by monitoring the total change in absorbance at 340 nm with a lactate dehydrogenase-coupled assay described in detail below.

### Purification of HOGA from bovine kidney

HOGA was purified from the cortex region of three freshly dissected bovine kidneys (88 g; Mitchells Meat Processing Company, Walnut Cove, NC) using a procedure adapted from Dekker *et al.* [15]. The tissue was resuspended in 900 mL of buffer A (225 mM mannitol, 75 mM sucrose, 5 mM MOPS pH 7.0, 0.1 mM EDTA, and four EDTA-free protease inhibitor tablets (Roche Diagnostics). The tissue was homogenized using a Brinkmann homogenizer (power setting 4.5) with 3 bursts, 30 sec each. Phenylmethylsulfonyl fluoride and benzamidine were added to a final concentration of 1 mM before centrifugation at 2,000 rpm for 20 min. The supernatant was decanted and the remaining liquid and pellet were then centrifuged at 8,500 rpm (JA-20 rotor) for 10 min. The pellets were washed with buffer A three more times and then centrifuged at 18,000 rpm for 20 min, yielding ∼12 g of purified mitochondria that were stored at −80°C. Six grams of mitochondria in buffer A were heated to 70°C, and the resulting precipitate was removed by centrifugation at 18,000 rpm for 20 min. The supernatant was processed using AmSO_4_ fractionation (20–60% cuts in 10% increments) in buffer B (50 mM TRIS pH 7.8 and 2.5 mM dithiothreitol (DTT)). The phenylhydrazine assay was used to identify that the 30–40% (w/v) AmSO_4_ fractions contained HOGA activity, *i.e.* formed glyoxylate from HOG [21]. These sample pools were dialyzed into buffer B and fractionated using a DEAE Macro-Prep column (Bio-Rad) and a 0–500 mM NaCl gradient over 900 mL. Those fractions containing the highest HOGA activity were pooled, concentrated to 5 ml using a VivaSpin20 device with a 10 kDa cutoff, and loaded onto a Superdex 200 size-exclusion column (GE Healthcare) equilibrated with 50 mM TRIS pH 7.8, 100 mM NaCl, and 2.5 mM DTT. HOGA was concentrated to 2.1 mg ml^−1^, aliquoted, frozen with liquid N_2_, and stored at −80°C.

### Identification of bovine and human HOGA genes by mass spectrometry

Bovine HOGA was submitted to the W. M. Keck Foundation Biotechnology Resource Laboratory (New Haven, CT) for analysis. In summary, the protein was denatured by the addition of 8 M urea/0.4 M NH_4_HCO_3_. The Cys residues were blocked by the addition of iodoacetamide. Trypsin was added at a 1∶15 wt/wt ratio (Promega V5111) and the volume was increased to 80 µL using H_2_O. The sample was digested at 37°C for 18 hr. LC-MS/MS data were collected on a Waters Q-Tof Ultima mass spectrometer and analyzed by MASCOT (Matrix Science Ltd.).

### Recombinant expression and purification of human HOGA variants

The human HOGA gene (*HOGA1*) was subcloned into the pET151/D-TOPO vector (Invitrogen) so that the mitochondrial targeting sequence was removed (i.e., the protein started at residue 26). In addition, this construct contained an N-terminal His-tag and an engineered, intervening PreScission Protease recognition site (GE Healthcare) to facilitate removal of the affinity tag. The S77A, S77T, S77V, N78A, N78T, N78Q, Y140F, Y168F, K196A, S198A, and S198T mutants of hHOGA were made using the QuikChange site-directed mutagenesis procedure (Stratagene).

The wild-type (WT) and the Y140F, Y168F, and K196A hHOGA variants were overexpressed in 6 L cultures of BL21*(DE3) *Escherichia coli* at 37°C. The culture was induced by the addition of 0.1 mM isopropyl-β-D-thio-galactoside (IPTG) once the OD_600_ reached 0.6–0.8, and the temperature decreased to 16°C overnight. The cells were resuspended in 100 mL of buffer C (50 mM HEPES pH 7.9, 500 mM KCl, 0.1% Triton X-100, 10% glycerol, 0.5 mM imidazole, 1 mM PMSF and benzamidine), lysed using an Avestin Emulsiflex-C5 cell homogenizer, and loaded onto a nickel nitrilotriacetic acid affinity column (Qiagen). The column was sequentially washed with several column volumes of buffer C and buffer C that did not contain the Triton X-100, glycerol, and protease inhibitors. HOGA was eluted with a 5–250 mM imidazole gradient. The desired fractions, as determined by SDS-PAGE analysis, were pooled, dialyzed into 20 mM HEPES pH 8.5, and fractionated on a Q-Sepharose HP column (GE Healthcare) with a 0–0.5 M NaCl gradient over 300 mL. The fractions containing HOGA were further purified via passage over a Superdex 200 size-exclusion column equilibrated with 20 mM HEPES pH 7.5, 100 mM NaCl. The protein was readily concentrated to 10–20 mg mL^-1^, aliquoted, frozen on liquid N_2_, and stored at −80°C. For experiments with the protein without the His-tag, the protein was treated with PreScission Protease (50∶1 ratio, 15 mM DTT; GE Healthcare) overnight at 4°C prior to the anion exchange column step. Removal of the His-tag was confirmed by mass spectrometry. The molecular masses of the hHOGA with and without the His-tag are 36,136 Da and 33,097 Da, respectively. The His-tag free protein does contain three additional N-terminal residues (GPH) that were originally part of the PreScission Protease recognition site.

In an effort to overcome low solubility and poor overall yields, some hHOGA variants (S77A, S77T, S77V, N78A, N78T, N78Q, S198A, and S198T) were subcloned into the pMal expression vector (New England BioLabs). In this process, an intervening PreScission Protease cleavage sequence was engineered. The expression conditions and purification scheme were modified as follows. The BL21-Gold (DE3) *E. coli* cells were induced with 0.3 mM IPTG. The cells were resuspended with 20 mM TRIS pH 7.5, 200 mM NaCl and lysed. The cleared cell lysate was first passed over an amylose column (New England Biolabs) pre-equilibrated with 20 mM TRIS pH 7.5, 200 mM NaCl. Bound MBP-hHOGA was eluted off the column via the addition of 10 mM maltose. Overnight dialysis exchanged the buffer to 20 mM TRIS pH 8.5, 0.5 mM dithiothreitol (DTT), and 0.1 mM EDTA. PreScission Protease (50∶1) was added to cleave MBP from HOGA during dialysis at 4°C. Removal of the MBP-tag was confirmed by mass spectrometry. A Q-Sepharose HP column was used to separate cleaved MBP from hHOGA. Glutathione S-Sepharose was also used to remove any remaining PreScission Protease. The remaining size-exclusion, concentration, and storage parameters were the same as described as above. Typical yields for all proteins were 10–30 mg from a 6 L culture.

The global folding of each hHOGA variant was evaluated by triplicate circular dichroism (CD) spectra that were averaged from 200–250 nm at 25°C, using a cuvette with a 0.05 cm path length on a Jasco 720 spectrometer. The proteins were diluted to 0.2 mg mL^−1^ in 20 mM HEPES pH 7.5, 100 mM NaCl. Data are presented as molar ellipticity *vs.* wavelength.

### Measurement of HOGA activity

A standard lactate dehydrogenase (LDH) coupled-enzyme assay was used to assess the activity of hHOGA by monitoring the production of pyruvate from the cleavage of HOG. The decrease in NADH absorbance at 340 nm was continuously monitored using a Cary50 spectrophotometer. The reaction rates were converted to M s^−1^ using the extinction coefficient for NADH (ε = 6220 M^−1^ cm^−1^). The 200 µL reactions contained 10–100 nM hHOGA, 200 µM NADH, 100 mM TRIS pH 8.5, 200 mU LDH (Sigma; Rabbit muscle Type 2), and 4.7–400 µM HOG. The reactions were run at 37°C in triplicate and repeated on several different days with fresh aliquots of hHOGA and HOG. The *k*_cat_, *K*_M_, and *k*_cat_ */K*_M_ values for HOG were determined utilizing nonlinear regression analysis in the enzyme kinetics module of SigmaPlot 11.0 (Systat Software, San Jose, CA).

### Oligomeric state analyses

Three different techniques were used to evaluate the oligomeric state of hHOGA. First, as a part of the purification process, hHOGA was passed over a calibrated Superdex 200 size-exclusion column. The calibrants used were blue dextran (2,000 kDa), aldolase (158 kDa), bovine serum albumin (67 kDa), ovalbumin (43 kDa), and ribonuclease A (12.7 kDa) (GE Healthcare). Second, sedimentation equilibrium analytical ultracentrifugation data for three protein concentrations and two rotor speeds (7,500 and 10,500 *g*) were collected using a Beckman Optima XL-A instrument using 12 mm double sector cells with quartz windows and an An60-Ti analytical rotor. The analyses were performed on both the His-tag- and MBP-free versions of hHOGA diluted in 20 mM HEPES pH 7.5, 100 mM NaCl. The protein concentrations for these experiments were as follows: experiment #1, 0.43, 0.82, 1.25 mg ml^−1^; experiment #2, 0.46, 0.78, 1.14 mg ml^−1^. Data were analyzed globally using the HETEROANALYSIS package (Version 1.1.28, J.W. Cole and J.W. Lary, Analytical Ultracentrifugation Facility, Biotechnology/Bioservices Center, University of Connecticut, Storrs, CT). The partial specific volume of 0.7396 cm^3^ g^−1^ (calculated from amino acid composition), solvent density of 1.0039 g cm^−3^ (measured with a Mettler DA310 Density/Specific Gravity Meter), and a single ideal species model were used to determine the weight-average molecular weight for each preparation. The data were also fit to monomer-dimer-tetramer and dimer-tetramer equilibria using HETEROANALYSIS and SEDPHAT (Version 8.2) [30]. Third, dynamic light scattering data were collected on 0.25–25 mg mL^−1^ hHOGA solutions in the same buffer above using a Malvern Zetasizer NanoS instrument.

### Human HOGA crystallization

Crystals of HOGA were obtained by the vapor diffusion method. Equal volumes of protein (12 mg mL^−1^ in 20 mM HEPES pH 7.5, 100 mM NaCl) and well solution (18% PEG 3350, 200 mM KSCN, 100 mM Bis-tris propane pH 7.5, 15 mM TCEP, 25% ethylene glycol) were mixed and incubated at 20°C for 3–7 days as hanging drops. For the pyruvate bound hHOGA crystals, 10 mM sodium pyruvate was added to the protein solution prior to setting up the crystal trays. For the ethylmercuriothiosalycilate (EMTS)-derivatized hHOGA crystals, 500 µM EMTS was incubated with hHOGA at 4°C overnight. Excess EMTS was removed via a Bio-Rad P-6 desalting column before setting up crystallization experiments.

### Data collection and structure determination

A single-wavelength anomalous dispersion (SAD) dataset was collected using an in-house Rigaku/MSC MicroMax-007 generator with a Saturn-92 CCD detector on EMTS-containing crystals of hHOGA. X-ray diffraction data for the apo hHOGA crystals were collected on beamline X25 (λ = 1.5 Å) at the National Synchrotron Light Source (Upton, NY) using an ADSC Quantum-4 CCD detector. For the pyruvate bound hHOGA crystals, data were collected in-house using a Rigaku/MSC RU-H3R generator with an R-Axis IV image plate detector. All datasets were processed with d*Trek (Rigaku/MSC, The Woodlands, TX) [53]. All crystals exhibited P6_4_22 symmetry (*a* = *b* = 141 Å, *c* = 108 Å) with one molecule in the asymmetric unit. PHENIX AutoSol was used to detect the anomalous signal from the heavy atom dataset and generate initial electron-density maps [54]. A total of 23 EMTS sites were found with occupancies ranging from 0.07–1.0, three of which were used for phase determination. An initial FOM value of 0.51 was obtained and improved to 0.71 following density modification. The AutoBuild routine within PHENIX was able to build 97% of the structure. After several rounds of refinement using CNS, the heavy-atom based model was used as a molecular replacement search model within PHASER for the uncomplexed and pyruvate bound hHOGA datasets [31]. All models were initially refined with CNS using alternating cycles of simulated-annealing, positional, and B-factor refinement [55]. Model building was performed with COOT [56]. Water molecules were identified with a *F_o_–F_c_* map contoured at 3 σ and added with COOT. Several ethylene glycol molecules were also added. The final cycles of refinement were performed with REFMAC5 [57]. The structures were validated using the MolProbity server, which reported 96.9–98% of the residues present in the Ramachandran favored regions [58]. The data collection and refinement statistics are summarized in Table 1. All structural figures were generated with PyMOL (The PyMOL Molecular Graphics System, Version 1.3, Schrödinger, LLC.). The coordinates and structure factors for the apoenzyme (PDB ID: 3S5N) and pyruvate-Schiff base complex (PDB ID: 3S5O) have been deposited to the Protein Data Bank (PDB).

## Supporting Information

Figure S1 **Quaternary structure and pyruvate complexes of representative KDPGA and DHDPS enzymes.** (A) Trimeric organization of *T. maritima* KDPG aldolase (PDB ID: 1WA3) [24]. (B) Pyruvate complex of *T. maritima* KDPGA. The pyruvate molecule (cyan) is covalently attached as a Schiff base to Lys129 and interacts with Arg17. A hydrogen-bonding network includes intervening water molecules, Glu40, Lys129, and Thr156. The adjacent monomer contributes two amino acids (Ala143′ and Pro147′) near this region and binds a sulfate ion, most likely mimicking the phosphate binding site for these enzymes. (C) Tetrameric organization of *E. coli* DHDPS (PDB ID: 3DU0) [28]. The tetramer consists of a dimer of dimers. (D) Active site of *E. coli* DHDPS. The pyruvate molecule (cyan) is covalently attached as a Schiff base to Lys161 (green) and interacts with Thr45. A hydrogen-bonding network is formed between Thr44, Tyr133, and Tyr107′ from the adjacent monomer (gray).
(TIF)

Figure S2 **Size exclusion chromatography analysis of hHOGA molecular weight.** Protein standards (blue diamonds) were used to calibrate a Superdex 200 gel filtration column: blue dextran (2,000 kDa) aldolase (158 kDa), bovine serum albumin (67 kDa), ovalbumin (43 kDa), ribonuclease A (12.7 kDa) (GE Healthcare). The elution position (V_e_), column void volume (V_o_), and the bed volume of the column (V_t_) were used to calculate K average, defined as ([(V_e_-V_o_)/V_t_-V_o_)]), and plotted against the logMW. The theoretical elution position for the monomeric, dimeric, trimeric, and tetrameric forms of hHOGA are shown as black squares. The elution position of recombinant hHOGA is indicated by the green triangle.
(TIF)

Figure S3 **Fit of the sedimentation equilibrium analytical centrifugation data to monomer-dimer-tetramer and dimer-tetramer models.** Data obtained at 3 concentrations and 2 rotor speeds were analyzed using the different equilibria models and the HETEROANALYSIS and SEDPHAT algorithms. The fits for the dimer-tetramer and monomer-dimer-tetramer models are shown in the sold and dotted lines, respectively. The calculated K_d_ values for the dimer-tetramer equilibrium ranged from 58–63 µM.
(TIF)

Figure S4 **Dynamic light scattering analysis of hHOGA.** Data were collected on a Malvern Zetasizer NanoS instrument using a hHOGA concentration range from 0.25–25 mg mL^-1^. Inset: Plot of peak diameter versus protein concentration. The theoretical diameter for the monomer, dimer, trimer, and tetramer of hHOGA, assuming a spherical globular shape, are indicated by the black squares.
(TIF)

Figure S5 **Experimental electron density map and unit cell packing.** (A) Placement of the initial hHOGA model into the 2.10 Å resolution, Hg^2+^-phased experimental electron density map contoured to 1.5 σ (light blue). A contour of the map to 5 σ (green) highlights the Hg^2+^ atom bound to Cys139. Atom colors are as follows: light-orange, carbon atoms; blue, nitrogen; red, oxygen; gray, mercury. (B) Orthogonal views of hHOGA packing within the unit cell exhibiting P6_4_22 symmetry. Each representative tetramer is shown in shades of a different color. The asymmetric unit (a.s.u.) is outlined with a dashed box and contains one monomer.
(TIF)

Figure S6 **Representative kinetic analyses for the wild-type and Y140F variants of hHOGA.**
(TIF)
