# Supplementary figures and images for: Structural and Biochemical Studies of Human 4-hydroxy-2-oxoglutarate Aldolase: Implications for Hydroxyproline Metabolism in Primary Hyperoxaluria
Source: PLoS One. 2011 Oct 6;6(10):e26021. doi: 10.1371/journal.pone.0026021 (PMC3188589; doi:10.1371/journal.pone.0026021)

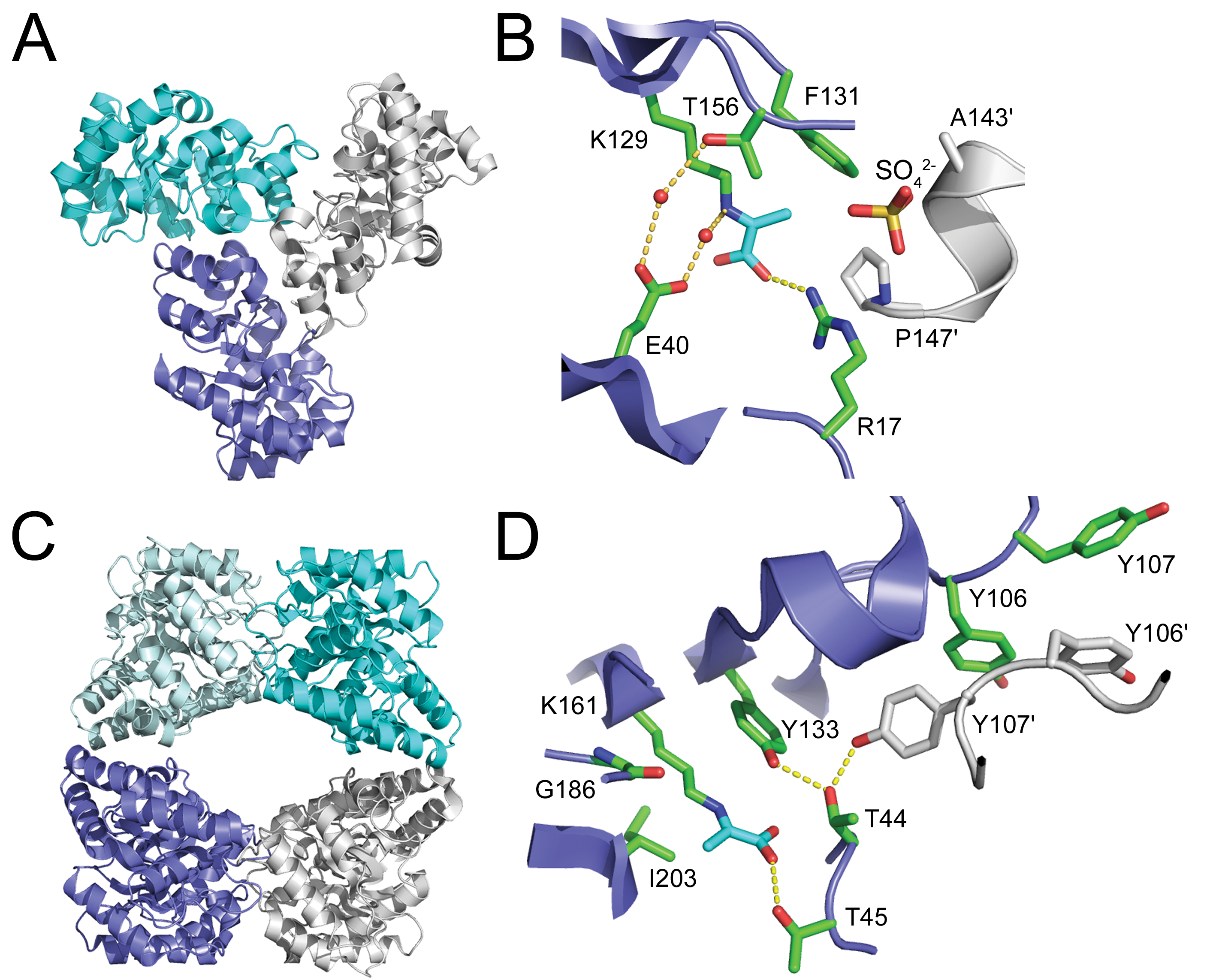

Supplement: Figure S1 — Quaternary structure and pyruvate complexes of representative KDPGA and DHDPS enzymes. (A) Trimeric organization of T. maritima KDPG aldolase (PDB ID: 1WA3) [24]. (B) Pyruvate complex of T. maritima KDPGA. The pyruvate molecule (cyan) is covalently attached as a Schiff base to Lys129 and interacts with Arg17. A hydrogen-bonding network includes intervening water molecules, Glu40, Lys129, and Thr156. The adjacent monomer contributes two amino acids (Ala143′ and Pro147′) near this region and binds a sulfate ion, most likely mimicking the phosphate binding site for these enzymes. (C) Tetrameric organization of E. coli DHDPS (PDB ID: 3DU0) [28]. The tetramer consists of a dimer of dimers. (D) Active site of E. coli DHDPS. The pyruvate molecule (cyan) is covalently attached as a Schiff base to Lys161 (green) and interacts with Thr45. A hydrogen-bonding network is formed between Thr44, Tyr133, and Tyr107′ from the adjacent monomer (gray). (TIF) [file pone.0026021.s001.tif]

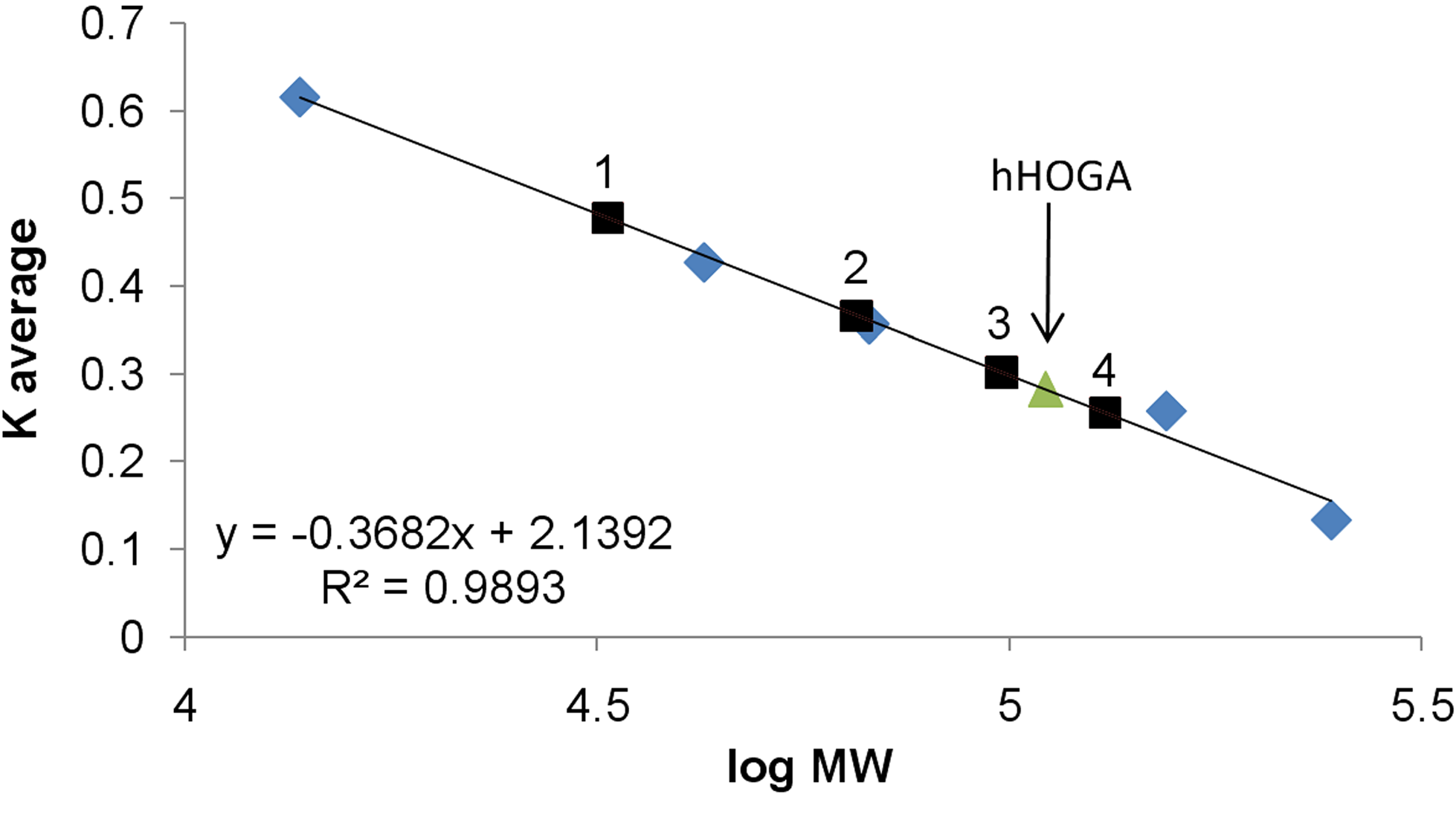

Supplement: Figure S2 — Size exclusion chromatography analysis of hHOGA molecular weight. Protein standards (blue diamonds) were used to calibrate a Superdex 200 gel filtration column: blue dextran (2,000 kDa) aldolase (158 kDa), bovine serum albumin (67 kDa), ovalbumin (43 kDa), ribonuclease A (12.7 kDa) (GE Healthcare). The elution position (Ve), column void volume (Vo), and the bed volume of the column (Vt) were used to calculate K average, defined as ([(Ve-Vo)/Vt-Vo)]), and plotted against the logMW. The theoretical elution position for the monomeric, dimeric, trimeric, and tetrameric forms of hHOGA are shown as black squares. The elution position of recombinant hHOGA is indicated by the green triangle. (TIF) [file pone.0026021.s002.tif]

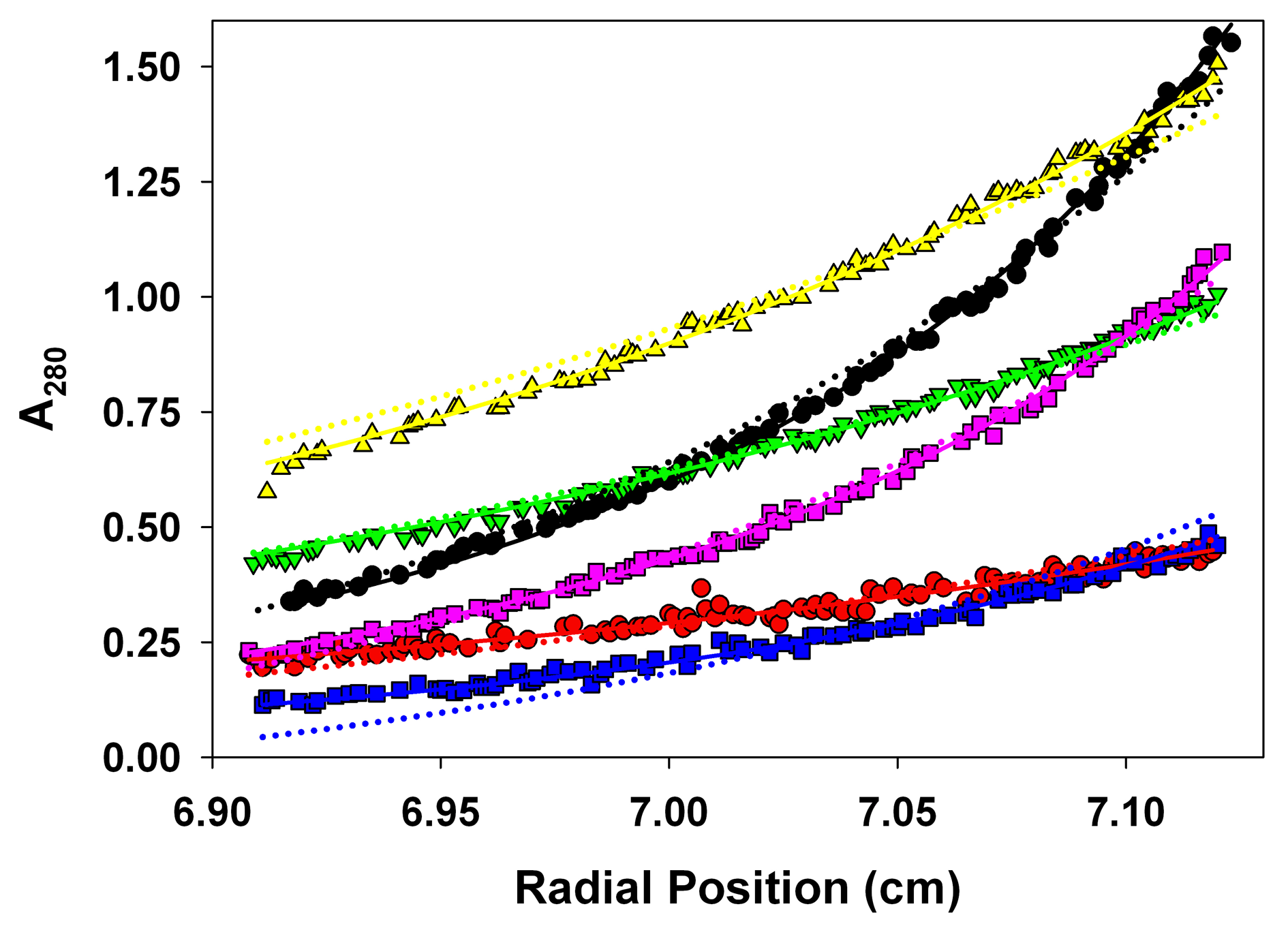

Supplement: Figure S3 — Fit of the sedimentation equilibrium analytical centrifugation data to monomer-dimer-tetramer and dimer-tetramer models. Data obtained at 3 concentrations and 2 rotor speeds were analyzed using the different equilibria models and the HETEROANALYSIS and SEDPHAT algorithms. The fits for the dimer-tetramer and monomer-dimer-tetramer models are shown in the sold and dotted lines, respectively. The calculated Kd values for the dimer-tetramer equilibrium ranged from 58–63 µM. (TIF) [file pone.0026021.s003.tif]

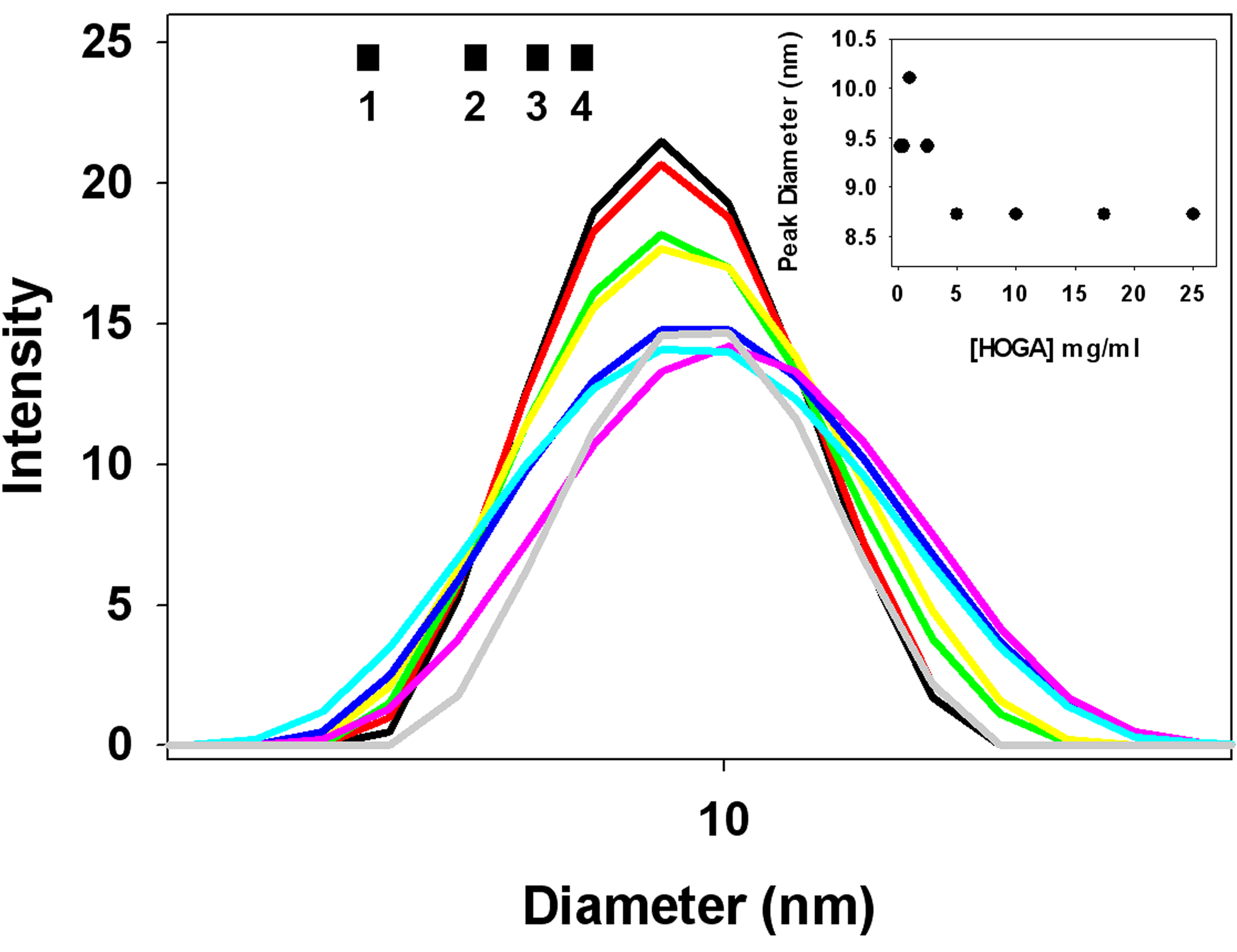

Supplement: Figure S4 — Dynamic light scattering analysis of hHOGA. Data were collected on a Malvern Zetasizer NanoS instrument using a hHOGA concentration range from 0.25–25 mg mL-1. Inset: Plot of peak diameter versus protein concentration. The theoretical diameter for the monomer, dimer, trimer, and tetramer of hHOGA, assuming a spherical globular shape, are indicated by the black squares. (TIF) [file pone.0026021.s004.tif]

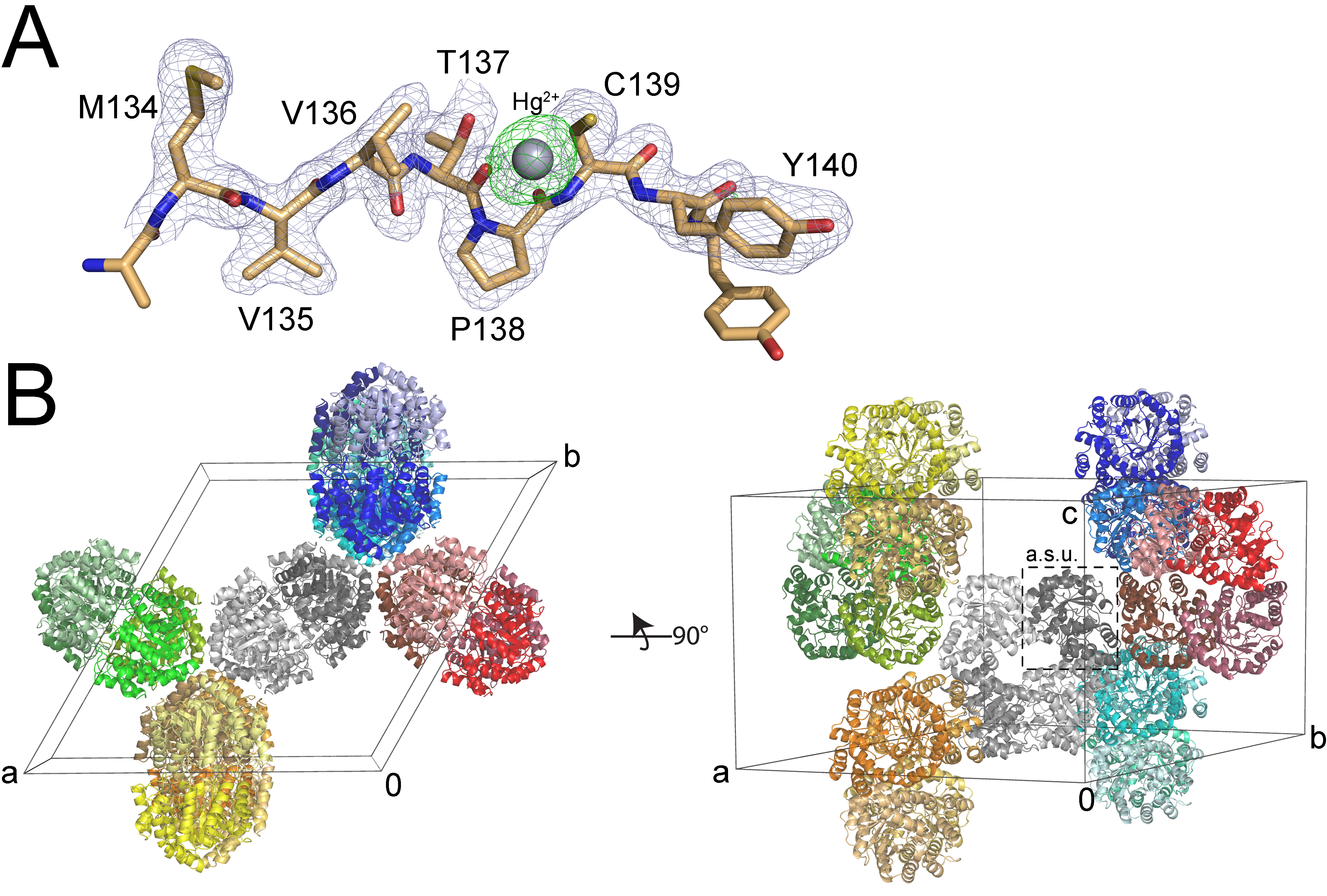

Supplement: Figure S5 — Experimental electron density map and unit cell packing. (A) Placement of the initial hHOGA model into the 2.10 Å resolution, Hg2+-phased experimental electron density map contoured to 1.5 σ (light blue). A contour of the map to 5 σ (green) highlights the Hg2+ atom bound to Cys139. Atom colors are as follows: light-orange, carbon atoms; blue, nitrogen; red, oxygen; gray, mercury. (B) Orthogonal views of hHOGA packing within the unit cell exhibiting P6422 symmetry. Each representative tetramer is shown in shades of a different color. The asymmetric unit (a.s.u.) is outlined with a dashed box and contains one monomer. (TIF) [file pone.0026021.s005.tif]

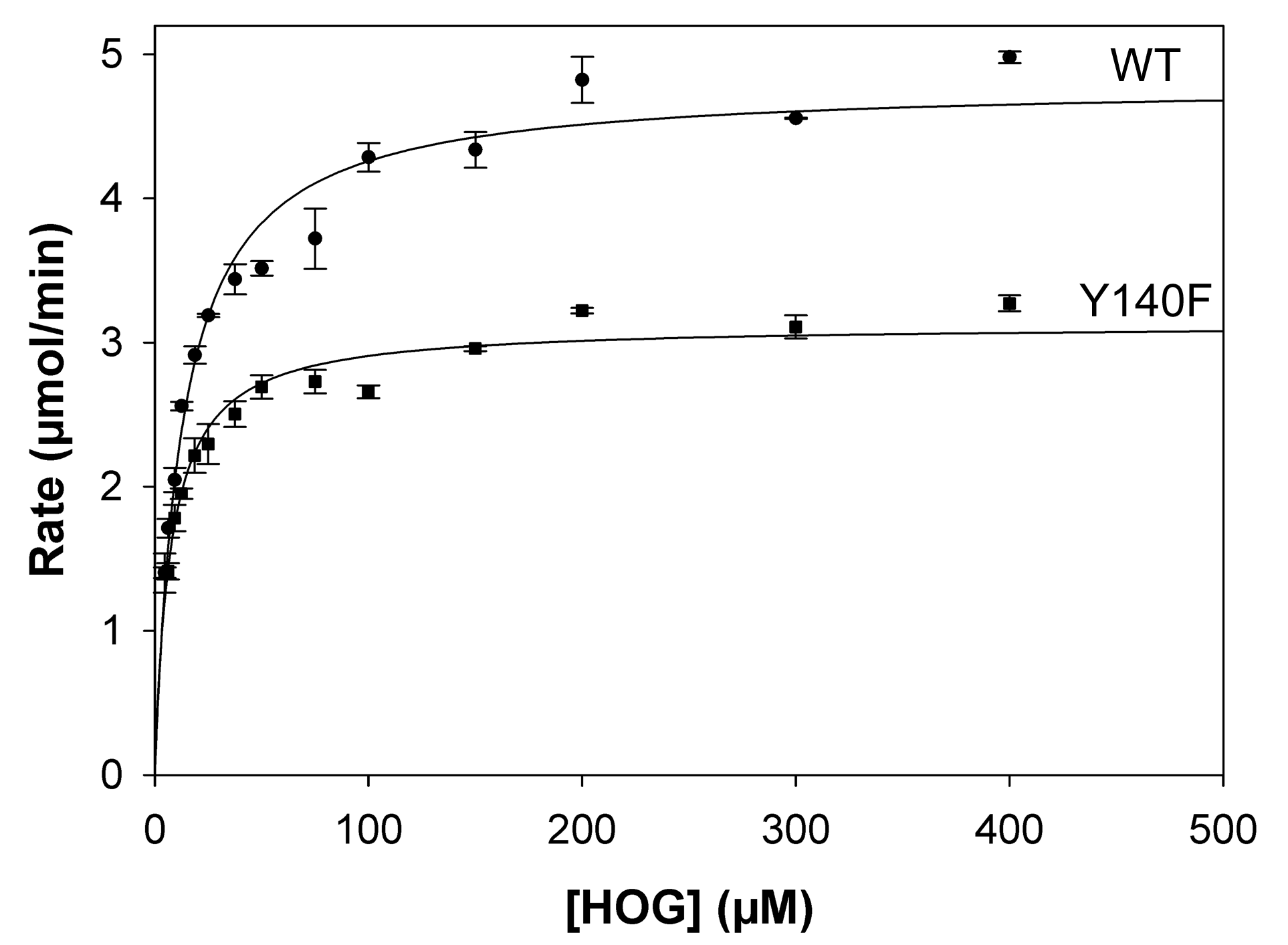

Supplement: Figure S6 — Representative kinetic analyses for the wild-type and Y140F variants of hHOGA. (TIF) [file pone.0026021.s006.tif]
